# *Allium* Flavonols: Health Benefits, Molecular Targets, and Bioavailability

**DOI:** 10.3390/antiox9090888

**Published:** 2020-09-19

**Authors:** Damini Kothari, Woo-Do Lee, Soo-Ki Kim

**Affiliations:** Department of Animal Science and Technology, Konkuk University, Seoul 05029, Korea; damini.kth@gmail.com (D.K.); caw147@naver.com (W.-D.L.)

**Keywords:** *Allium*, flavonols, health benefits, antioxidant effects, molecular targets, bioavailability

## Abstract

*Allium* species are revered worldwide as vegetables, condiments, and spices as well as the therapeutic agents in traditional medicine. The bioactive compounds in alliums mainly include organosulfur compounds, polyphenols, dietary fibers, and saponins. Flavonoids, particularly flavonols from alliums, have been demonstrated to have the antioxidant, anticancer, hypolipidemic, anti-diabetic, cardioprotective, neuroprotective, and antimicrobial activities. However, flavonols are mostly characterized from onions and have not been comprehensively reviewed across different species. This article therefore focuses on flavonol profiles from different *Allium* species, their health effects, underlying molecular mechanisms, and bioavailability. Intriguingly, the functional health effects of flavonols were mainly ascribed to their antioxidant and anti-inflammatory activities involving a cascade of multiple signaling pathways. Although the *Allium*-derived flavonols offer tremendous potential in preventing chronic disease risks, in-depth studies are needed to translate their clinical application.

## 1. Introduction

Chronic diseases pose a huge burden on global health care system and economy accounting for nearly seventy percent of pre-mature mortality en masse [1]. According to the World Health Organization (WHO), poor dietary habits which include consumption of foods with low-nutrient density and high in fat, sugar, and salt as well as overall calories, are one of the major contributors to the leading causes of chronic illness and related deaths [1,2]. We emphasize that the dietary co-administration of the antioxidant-rich foods along with clinical treatments may ameliorate chronic illnesses mediated by heightened free radicals in the body. It has particularly been observed that people with chronic illnesses are more vulnerable to clinical complications and deaths by novel severe acute respiratory syndrome coronavirus (SARS-CoV-2) infections [3,4,5]. The on-going coronavirus disease (CoVID-19) pandemic has forced people across the globe to rethink their nutritional habits, switching to antioxidant-rich foods to avoid chronic illness with stronger immunity [6,7]. Antioxidant-rich foods reportedly enhance the T-cell subsets, interleukin production, and natural killer cells necessary to ward off influenza-like infections [7,8]. Regular consumption of *Allium* herbs is traditionally considered beneficial for human health owing to their rich contents of antioxidant compounds. The genus *Allium* (Amaryllidaceae) is one of the largest monocot genera comprising approximately 915 species including culinary herbs like garlic, onion, shallot, leek, chives, and scallions [9]. The main bioactive constituents in alliums include organosulfur compounds (OSCs) and polyphenols [10]. However, the complex chemistry of OSCs, due to their highly-volatile and thermally-unstable nature, likely impairs their health benefits [10,11,12,13]. In this context, allium polyphenols, with relatively higher stability than OSCs and associated antioxidant functions have emerged as more viable bioactive constituents in *Allium* species. A range of health-modulating effects of *Allium*-derived polyphenols, in particular, flavonols, are attributed to their antioxidant activity which governs their functional properties including anti-inflammatory, antimicrobial, antiglycemic, and anticancer effects [14,15,16]. Though various *Allium* species are rich source of dietary flavonols, most studies have focused on the onion. The present review highlights the current knowledge about well-characterized flavonols from different *Allium* spp., their established health effects, and the associated subtle mechanisms based on their antioxidant properties as well as bioavailability.

## 2. Flavonoids in *Allium*: Structural Properties

**Figure 1:**
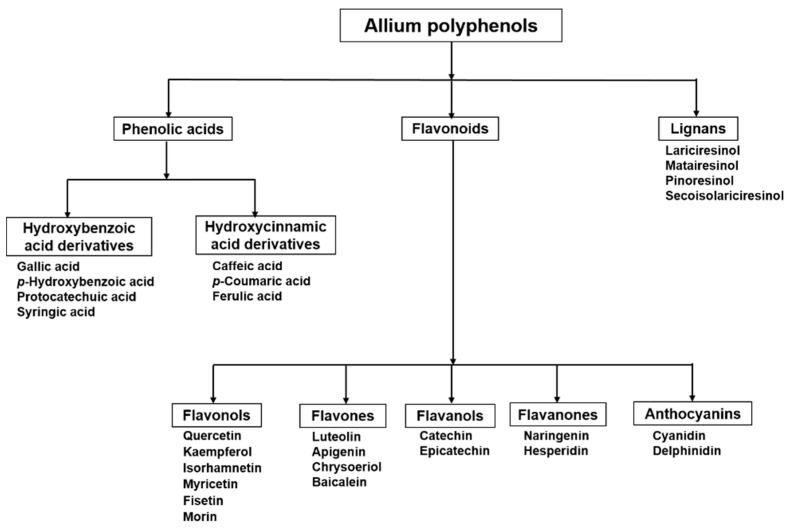
Polyphenolic compounds in the genus *Allium*.

The major polyphenolic compounds in alliums include flavonoids, phenolic acids, and lignans (http://phenol-explorer.eu/) [17] (Figure 1). Flavonoids are the largest class of polyphenols followed by phenolic acids in alliums, while lignans are a minor polyphenol class [17]. Flavonoids have a characteristic 15-carbon (C6-C3-C6) skeleton, which consists of two phenyl rings (A and B) and a heterocyclic ring (C) [18,19]. They are classified into different subclasses (flavonols, flavanols, flavanones, flavones, anthocyanins, and isoflavones) based on the degree of unsaturation and oxidation of the C-ring. Among different subclasses, further division is based on the number and nature of substituent groups attached to their heterocyclic rings [18,19]. Flavonols and anthocyanins are the main subclasses of flavonoids present in alliums, though the latter have been found only in red onions [20,21]. Notably, the flavonol content in different *Allium* spp. ranges from 7 to 1917 mg/kg fresh weight (Table 1). Herein, we intended to give a comprehensive detail of the flavonol constituents and related bioactivities from alliums.

### Flavonols

**Figure 2:**
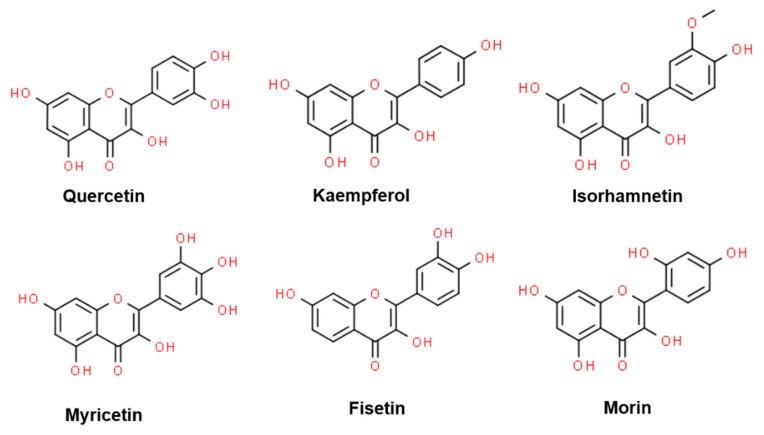
Chemical structure of the major representatives of flavonol aglycones in *Allium* spp.

Flavonols, the most abundant class of flavonoids in alliums [22,23,24], contain a 3-hydroxyflavone backbone (IUPAC name: 3-hydroxy-2-phenylchromen-4-one) [18,19]. Their diversity arises from the different positions of the phenolic -OH groups. Quercetin, kaempferol, isorhamnetin, myricetin, fisetin, and morin are the major flavonol aglycone representatives in alliums (Figure 2). At least 52 different kinds of flavonols have been identified from different *Allium* spp. (Table 2). Most of these flavonols exist as their glycosylated derivatives in nature, where sugars are attached through oxygen at 3, 4′, and/or 7-positions of the aglycones. Glucose is the most common sugar moiety; however, rhamnose, galactose, xylose, and glucuronic acid have also been identified (Table 2). Generally, flavonol glycosides have significantly lower antioxidant capacity than their respective aglycones [31]. Chemically, the antioxidant activity of flavonols is owed to their phenolic hydroxyl group via donation of hydrogen atom to free radicals [32]. The hydroxyl groups in B-ring (4′-position) and C-ring (3-position) have higher hydrogen donation ability than the A-ring hydroxyl groups [31]. The concentration (Table 1) and type (Table 2) of flavonols vary widely among species, cultivars, and parts of the plant. In addition, post-harvest practices [33] and seasons [34] are also reported to affect the flavonol concentration in alliums. Major flavonols found in onion cultivars are the derivatives of quercetin while kaempferol and isorhamnetin derivatives are lesser abundant [22,23,24]. Quercetin 3,4′-glucoside (3,4′-Qdg) and quercetin 4′-glucoside (4′-Qmg) account for more than 90% of flavonols in onion [23,24]. In general, the levels of flavonols are higher in yellow onions than red onions [24]. Sweet onion contains two–three-fold higher isorhamnetin 4′-glucoside than red onion cultivars [35]. In chives, leeks, and scallions, kaempferol glycosides are the major flavonols [24,27,36]. Although several *Allium* flavonoids are characterized, the associated in vivo mechanisms are not fully understood owing to their cryptic catabolism following the dietary uptake. Below we discuss the health effects and associated functional mechanisms of *Allium*-derived flavonols based on the state-of-the-art literature and available metadata information.

## 3. Health Benefits of *Allium* Flavonols

### 3.1. Anticancer Effects

It has been widely accepted that dietary flavonoids lower cancer-related mortality [51,52,53]. Several pre-clinical studies evidenced that *Allium*-derived flavonols play an important role to delay cancer development and progression via multitude of mechanisms including anti-proliferation, induction of cell cycle arrest and apoptosis, as well as modulation of immunity and oxidative stress (Table 3). Flavonols extracted from *A. cepa* are reported to induce apoptosis in human leukemia cells (U937, THP-1, and K562) [16]. The onion extracts primarily contain eight flavonols including (1) quercetin 3,7,4′-triglucoside; (2) quercetin 7,4′-diglucoside; (3) quercetin 3,4′-diglucoside; (4) isorhamnetin 3,4′-diglucoside; (5) quercetin 3-glucoside; (6) quercetin 4′-glucoside; (7) isorhamnetin 4′-galactoside; and (8) isorhamnetin 4′-glucoside [16]. Considering its anticancer mechanisms, it has been observed that the onion flavonols activate caspases (caspase-3, -8, and -9) during apoptosis through both the cell death receptor (DR)-mediated extrinsic and mitochondria-mediated intrinsic pathways [16]. The extrinsic pathway involves the up-regulation of TNF-related apoptosis-inducing ligands (TRAIL) while the intrinsic pathway involves the downregulation of cellular anti-apoptotic proteins like cellular inhibitor of apoptosis protein-1 (cIAP-1), FLICE-like inhibitory protein (c-FLIP), and B-cell lymphoma extra large protein (Bcl-xL) [16]. A cross talk between the death receptor- and mitochondria-mediated caspase activation was also suggested by the authors following the flavonol interventions [16]. Reportedly, the onion extract triggers a molecular cascade leading to cancer cell apoptosis through extrinsic pathway, activating caspase 8 which promotes the cleavage of “Bid”, an apoptotic B-cell lymphoma-2 (Bcl-2) family protein [16]. The translocation of Bid to mitochondria results in the disruption of mitochondrial membrane potential (MMP, ΔΨm) and thus signals the release of cytochrome c (cyt c), and thereby initiating the mitochondria-mediated caspase activation [16]. In addition, the cancer cell apoptosis induced by onion-derived flavonols is partly mediated through the inhibition of phosphatidylinositol 3-kinase (PI3K)/Akt signaling pathway [16]. Nonetheless, how these two pathways are interrelated remains elusive.

Another seminal study by Lee et al. [54] reported that onion-derived flavonols trigger the “mitochondria mediated and caspase-dependent apoptosis” in AGS human gastric cancer cells through the inhibition of PI3K/Akt signaling pathways. Besides suppressing the Akt phosphorylation, the flavonols upregulate the p53 expression and subsequent Bax induction, resulting in reduced MMP (ΔΨm) and cyt c release, which is directly linked with the activation of caspase associated molecular cascade [54]. The authors also reported that the flavonol extracts also suppress the expression of mitochondria localized anti-apoptotic Bcl-2, a key regulator of apoptosis [54]. The anti-proliferative activities of Chinese onion (*A. chinense*), onion (*A. cepa*), and Welsh onion (*A. fistulosum*) extracts rich in quercetin glucosides (quercetin-3,4′-di-*O*-glucoside, 3,4′-Qdg and quercetin-4′-*O*-glucoside, 4′-Qmg) are also reported against liver, colon, and pancreatic cancer cell lines [55]. The authors observed a notably higher efficacy of 4′-Qmg toward the growth inhibition of cancer cells, as compared with 3,4′-Qdg. More recently, the subcritical water extracts of *A. hookeri* roots (30.6 mg of quercetin equivalent/g) are demonstrated to have a dose-dependent antiproliferative effects against fibrosarcoma and breast cancer cell lines [56]. During carcinogenesis, host immune system is often subverted by inflammation which in part due to oxidative stress [57]. The anti-inflammatory and immunomodulatory effects of flavonol-rich (quercetin and quercetin-4′-*O*-glucoside) red onion scale extract on prostate cancer rat model are observed by the suppressed expressions of pro-inflammatory molecules including interleukin (IL)-6, IL-8, and tumor necrosis factor-alpha (TNF-α) in prostatic tissues [14]. Furthermore, the attenuation of lipopolysaccharide (LPS)-induced oxidative as well as inflammatory stress in colon cancer cells (HT-29) has been reported by downregulating TNF-α expression and upregulating the expression of heme oxygenase-1 (HO-1) and glutathione *S*-transferase (GSTs) detoxification genes (i.e., GSTM1, GSTT1, and GSTP1) after onion peel extract (OPE) treatments [50]. Oxidative stress also acts a major stimulus of angiogenesis (the formation of new blood vessels from existing capillary networks) during tumor progression [58]. Seyfi et al. [59] demonstrated significant anti-angiogenic effects of flavonoid-rich *A. ascalonicum* (shallot) fraction in vitro, ex vivo, and in vivo models; however, the underlying mechanism is not investigated.

### 3.2. Anti-Obesity and Hypolipidemic Effects

Obesity is the underlying cause of several chronic diseases and is characterized by the excessive accumulation of adipose tissues (body fat) when energy intake surpasses the energy expenditure [60]. Obesity is a complex health issue that arises from a combination of causes and individual factors such as behavior and genetics. Behaviors can include physical activity, dietary patterns, medication use, and exposures to various environmental factors. Additional contributing factors include the food and physical activity environment, education and skills, and food marketing as well as promotion. *Allium* derived flavonol-rich extracts inhibit adipogenesis and intracellular lipid accumulation in cultured adipocytes and diet-induced obese animal models (Table 3). Several mechanistic studies suggested that quercetin-rich OPE induces lipolysis through the downregulated expression of transcriptional factors, including peroxisome proliferator-activated receptor-gamma (PPAR-γ) and CCAAT-enhancer binding protein alpha (C/EBPα) [61,62,63]. In addition, the genes associated with lipid metabolism including lipoprotein lipase (LPL), adipocyte fatty acid-binding protein (AFABP) and carnitine palmitoyltransferase 1α (CPT-1α) are also affected following OPE exposure [62,63]. Recent studies also have shown that autophagy plays an important role in adipogenesis and lipid metabolism. *Allium*-derived flavonols positively regulate autophagy via activation of AMP-activated protein kinase (AMPK) in cellular and animal models [64,65]. AMPK also plays an important role in the development and maintenance of brown and beige adipose tissue [66]. Recently, flavonol rich (quercetin and isoquercetin) OPE demonstrated browning effect on white adipose tissue through an AMPK-dependent pathway in mice adipose tissue and in cultured adipocytes [64]. The AMPK activation regulates the expression of brown adipocyte-specific genes such as uncoupling protein-1 (UCP-1), peroxisome proliferator-activated receptor gamma coactivator 1-α (PGC1α) and cell death-inducing DNA fragmentation factor-alpha-like effector A (CIDEA) [64].

The high lipid uptake into adipocyte in obese conditions often increases the mitochondrial substrate load, which subsequently increases electron transport chain activity and ROS-mediated oxidative stress. These enhanced ROS levels have been associated with increased insulin resistance [92]. Hence, it has also been argued that the anti-obesity effects of onion flavonols may be linked with reduced insulin resistance [93] and elevated blood antioxidant parameters [70,71,94] following the dietary interventions. Reportedly, quercetin and its glycoside derivatives isolated from onion waste improve lipid metabolism in HFD-fed rats through promoting the enzymatic activity of intestinal microbiota and the antioxidant capacity of blood [71]. In a follow-up study, the authors suggested a higher efficacy of 4′-Qmg in lowering plasma cholesterol and triglycerides than the 3,4′-Qdg in high-fat diet (HFD)-fed rats [94].

Some randomized controlled trials (RCTs) have also been investigated toward probing the effects of quercetin-rich *Allium* extracts on obesity in human subjects. In a study by Kim and Yim [70], OPE administration (100 mg/d, 50 mg bis a die) for 12 weeks significantly reduces waist and hip circumference, and the effects are ascribed to the reduced oxidative stress in obese women. However, the follow-up study showed no influence on the inflammatory mediators among the obese women [95].

### 3.3. Anti-Diabetic Effects

Type 2 diabetes is a worldwide epidemic and characterized by the elevated blood sugar levels due to impaired insulin action and/or secretion [96]. When diabetes is left unmanaged or untreated, increased blood glucose can damage the heart, blood vessels, eyes, kidneys, and nerves, leading to disability and premature death [96,97,98,99]. Several pre-clinical studies reported antidiabetic effects of flavonol-rich extracts from different *Allium* spp. in vitro [72,75] and in vivo [36,72,73] (Table 3). The ethanolic extract of onion skin (6.04 g quercetin/100 g dried weight of onion skin) is reported to lower postprandial blood glucose response in diabetic rats and the effects are ascribed to the inhibition of carbohydrate digestive enzymes including α-amylase, α-glucosidase, and sucrase [72]. Oxidative stress and inflammation are inter-linked and play a key role in the progression of type II diabetes [99,100,101,102]. Thus, the inhibition of oxidative stress might be an effective strategy to delay/prevent diabetes-related complications [103,104,105,106]. Jung et al. [73] reported that the supplementation of quercetin-rich OPE suppressed the biomarkers of serum oxidative stress (superoxide dismutase (SOD) activity and malondialdehyde (MDA) formation) and hepatic inflammation (TNF-α and IL-6), as well as improved the lipid profiles, levels of insulin receptors, and insulin-regulated glucose transporter type 4 (GLUT4) in HFD/streptozotocin (STZ)-induced diabetic rats. The antidiabetic mechanisms of flavonol may also be attributed to the insulin signal transduction through modulating the increased expression and phosphorylation of insulin receptors, insulin receptor substrate, and glucose transporter (GLUT) proteins [100]. Previously, Schulze et al. [75] also demonstrated the ability of onion extracts containing mainly the quercetin and its glucoside derivatives to inhibit intestinal “sodium-glucose linked transporter 1” (SGLT1) in vitro. Notwithstanding, the same study failed to elicit any hypoglycemic effects in vivo in normoglycemic mice and human volunteers, which might be attributed to some variations in dose efficacy or compound bioavailability.

Kaempferol is another important class of *Allium* flavonol which displayed promising antidiabetic potential [107,108,109,110]. A kaempferol-3-*O*-β-d-6(P-coumaroyl) glucopyranoside from onion displayed blood glucose lowering ability in alloxan-induced diabetic rats and it was comparably higher to a standard drug [74]. The authors suggested that the antidiabetic effect might be due to the beneficial effects of kaempferol glycoside on lipid metabolism and hepatic enzymes. Recently, kaempferol glycoside-enriched butyl alcohol fraction of *A. tuberosum* is also reported to reduce blood glucose levels via improving serum lipid profile and antioxidant parameters in alloxan-induced diabetic rats [36]. The antidiabetic effects of *Allium* flavonols are thought to be mediated by reducing blood glucose, serum lipids, oxidative stress, and lipid peroxidation, as well as increasing antioxidant enzyme activity and insulin secretion [36,72,73,74,76]. Nevertheless, the exact mechanism of hypoglycemic effects of *Allium*-derived flavonols is yet to be elucidated.

### 3.4. Cardio-Protective Effects

Cardiovascular disease (CVD) is the leading cause of deaths worldwide. A number of recent meta-analyses of prospective cohort studies have indicated that individuals with the highest flavonoid intakes have lower relative risk of mortality from CVD and all-causes compared with that of individuals with the lowest intakes [111,112,113]. Several studies evidenced the beneficial effects of *Allium* flavonols on the cardiovascular system via their regulatory effects on platelet aggregation [83,84,96], atherogenesis [78], thrombotic activities [79], endothelial function [81], hypertension [77,82], lipid metabolism [77], and oxidative stress [42] (Table 3). Ingestion of quercetin rich onion soup (69 mg total quercetin) inhibits platelet aggregation and essential components of the collagen-stimulated platelet activation pathway in human and may reduce the risk of thrombosis, a common pathology of CVD [114]. Dietary supplementation of onion powder containing high quercetin derivatives (50% 4′Qmg, 30% quercetin, and 20% 3,4′Qdg) attenuates the risk of atherosclerosis via lowering the atherogenic index and the incremental elastic modulus in rat models [78]. The oral supplementation of quercetin rich OPE (2 and 10 mg) demonstrates anti-thrombotic effects in a rat model [79]. The underlying mechanism of OPE-mediated anti-thrombosis involves the downregulated expression of thrombin-induced tissue factor, which is partly driven by the inactivation of mitogen-activated protein kinase (MAPK) signaling pathway, as observed by the reduced phosphorylation of extracellular signal-regulated kinase (ERK) and c-Jun N-terminal kinase (JNK) [79]. In another in vitro study by Ro et al. [83], quercetin-rich OPE inhibits collagen-induced rat platelet aggregation in a dose-dependent manner with IC_50_ value of 80.0 μg/mL. The anti-platelet aggregation effects of OPE has been ascribed to the reduced levels of intracellular Ca^2+^ and thromboxane A2 (TXA2) via regulation of cyclooxygenase-1 (COX-1), and TXA2 synthase (TXAS) activities. In addition, OPE also elevates cAMP levels, which might be related to the regulation of protein kinase activities [83]. Olayeriju et al. [42] showed that the ethyl acetate extract of red onion tunic affects hemodynamic parameters in rats. The active flavonols in the extract are identified as quercetin, quercitrin, isoquercitrin, rutin, and kaempferol, which might potentially reduce systolic and diastolic pressure, mean arterial blood pressure, pulse rate, and heart rate via modulating the oxidative stress [42].

Several human intervention studies have also demonstrated the efficacy of flavonol-rich onion in the reduction of CVD risks. Supplementary intake of onion extracts was found to lower blood pressure in smokers [77] and overweight-to-obese patients with pre-(hypertension) [82]. Endothelial function is another important predictor of cardiovascular events [80]. Chronic onion extract (rich in quercetin) intake ameliorates endothelial dysfunction through improving postprandial flow-mediated dilation (FMD) in healthy men [80]. Consumption of OPE (100 mg quercetin/d) for 12 weeks by healthy overweight and obese individuals is reported to improve endothelial function by increasing the FMD and circulating endothelial progenitor cell (EPC) counts [81]. However, not all flavonol-rich onion fractions demonstrate cardioprotective effects. Recently, Brüll et al. [115] observed no influence of onion skin extract (54 mg quercetin per capsule) on blood pressure, heart rate, or any biomarker of endothelial function in overweight-to-obese adults with hypertension.

### 3.5. Neuroprotective Effects

Neurodegenerative diseases, such as Alzheimer’s disease, Parkinson’s disease, frontotemporal dementia, Huntington disease, and amyotrophic lateral sclerosis are a growing burden worldwide due to their high prevalence yet poor treatment [116]. Oxidative stress and inflammation are the major contributors to the neurodegenerative disease [117,118,119]. The quercetin-rich *A. cepa* extract improves motor coordination and memory functions in aluminium chloride (AlCl_3_)-induced neurotoxicity in mice by ameliorating the tested oxidative stress biomarkers in serum including lipid peroxidation, glutathione levels, and catalase activity [89]. In addition, acetylcholinesterase (AChE) activity, a possible marker of low-grade systemic neuro-inflammation [120,121,122], is also significantly reduced in AlCl_3_ exposed animals receiving onion extract [89]. Previously, the kinetics studies of quercetin suggested its competitive type of inhibition against AchE [123]. Another study reported that the *A. cepa* outer scale extract (rich in quercetin) had positive effects on ischemia-reperfusion cerebral injury in a mice model by inhibiting lipid peroxidation and increasing antioxidant enzymes as well as antioxidants [91]. In addition, several in vitro studies investigated the mechanisms of neuroprotective effects of *Allium*-derived flavonols. Yang et al. [87] reported that onion derived quercetin protected hippocampal neuronal cells (HT22) against glutamate-mediated oxidative stress by reducing intracellular ROS production, Ca^2+^ influx, maintaining MMP, and downregulating several apoptosis-related biochemical markers. The onion flavonoids quench ROS in hypoxia-induced oxidative stress in neuronal cells via inactivation of protein kinase C-𝜀 (PKC-𝜀) and p38 mitogen-activated protein kinase (p38MAPK), induced by the phosphorylation of ERK1/2 [88]. The flavonoids from *A. victorialis* var. *platyphyllum* leaves and onion peel [124] were reported to exert neuroprotective effects in activated glial cells by inhibiting NO production, a pro-inflammatory mediator. These effects are ascribed to the strong antioxidant potential of flavonoids and their capacity to scavenge ROS [124]. A 3,4′-Qdg isolated from onion bulb protects human striatal precursor cells (HSPs) under nutrient deprived condition by reducing apoptosis and improving adhesion capacities via the expression of some adhesion molecules, such as pan-cadherin and focal adhesion kinase [90]. These studies indicate the potential application of *Allium*-derived flavonols in providing neuroprotective effects.

### 3.6. Antimicrobial Effects

In an antibiotic crisis world, flavonol-rich *Allium* extracts could act as a promising source of antimicrobial or antivirulence agents. Flavonol compounds exhibit antimicrobial activity against a broad range of antibiotic resistant pathogens in vitro. Recently, Sharma et al. [125] demonstrated antimicrobial activity of methanolic extract and isolated quercetin from onion against several multi-drug resistant (MDR) bacteria through in vitro and in silico experiments. The extract displayed higher zone of inhibition as compared with the isolated quercetin, which may be attributed to the synergistic effects of other co-existing bioactive compounds. The molecular docking analysis revealed that quercetin might interfere with the metabolism of bacterial proteins (β-lactamase, gyrase A, 2-trans-enoyl-acyl carrier protein reductase-inhA and topoisomerase IV) [125]. Snoussi et al. [46] demonstrated the in vitro antimicrobial potential of methanolic extracts of different plant parts from *A. roseum* var. *odoratissimum* against bacteria and yeast isolated from contaminated meat and sea foods. The authors attributed the observed antimicrobial effects of *Allium* extracts to the apigenin and kaempferol derivatives.

No matter how effective a new antimicrobial agent may be, pathogens will eventually develop resistance over time. Therefore, recently scientists lauded for studies on “antivirulence drugs” instead of “antimicrobial drug” to cope up with antibiotic resistance [126]. An antivirulence agent is not bactericidal per se but it attenuates the detrimental phenotypes of a pathogen and therefore, generally used as adjuncts to potentiate the existing antibiotic therapy [127]. Recently, Mahomoodally et al. [128] demonstrated that the polyphenol-rich extracts of onion and garlic augmented the efficacy of streptomycin and chloramphenicol against standard and clinical bacterial strains. The ability of 4′-Qmg from onion peel to inhibit biofilm formation regulated by quorum sensing (QS)-mechanisms and hence the virulence factors of *Chromobacterium violaceum* and *Pseudomonas aeruginosa*, has also been reported [129]. They established that 4′-Qmg treatment prevented the production of virulence factors (violacein, elastase, and pyocyanin) and biofilm formation in the test pathogens by antagonizing the virulence factor regulator (Vfr) and QS signal receptor (LasR) [129]. Another recent study witnessed the anti-QS activity of quercetin aglycone and 3-Qmg from onion on *C. violaceum*, *P. aeruginosa*, and *Serratia marcescens* by inhibiting violacein production and swarming ability [130]. The above discussion imparts that the use of *Allium*-derived flavonols could be a sustainable solution to mitigate issues relating to the resistant microbial infections. However, we emphasize further in vivo studies and clinical trials prior to any formal promotion of flavonols toward disease management involving MDR pathogens.

### 3.7. Other Health Benefits

Immunomodulatory effects of flavonoid-rich fraction of *A. ascalonicum* (shallot) extract is reported via inducing delayed-type hypersensitivity and TH1 cytokine-IFNγ expression in a mouse model [131]. *A. mongolicum* extract (100 and 200 mg/kg) containing flavonols (quercetin, kaempferol, and their respective glucosides) has been reported to induce the recovery of fecal excretion and water content while enhancing colon thickness and number of goblet cells in a loperamide (LOP)-induced constipated mouse model [132]. The anti-constipation effects are mediated by the downregulation of aquaglyceroporin-3 (AQP3) expression and up-regulation and activation of G protein alpha (Gα) and phosphoinositide 3-kinases (PI3K) [132]. Cho et al. [133] reported the protective effects of onion extract (quercetin; 17.1 μM) against radiation-induced cyto- and geno-toxicity in human lymphocytes pertaining to its antioxidant and DNA repair properties.

The *A. tuberosum* extracts containing glycosides of quercetin, kaempferol, and isorhamnetin demonstrated antiviral effects in ovo against an avian influenza (low-pathogenic) virus [40]. The antiviral efficacy of flavonols is attributed to their ability to interact with various molecular targets [134,135,136]. Quercetin derivatives are reported to target cap-binding site of PB2 of influenza viral RNA polymerase [134]. Schwarz et al. [136] found that kaempferol derivatives block virus ion channels in SARS coronavirus. A recent molecular docking analysis revealed that fisetin, kaempferol, and quercetin target SARS-CoV2-S spike protein of CoVID-19 [135]. These studies indicate that *Allium*-derived flavonols may present a promising and relevant therapeutic option for the management of CoVID-19 infection.

## 4. Molecular Mechanisms Underlying the Physiological Effects of Flavonols

**Figure 3:**
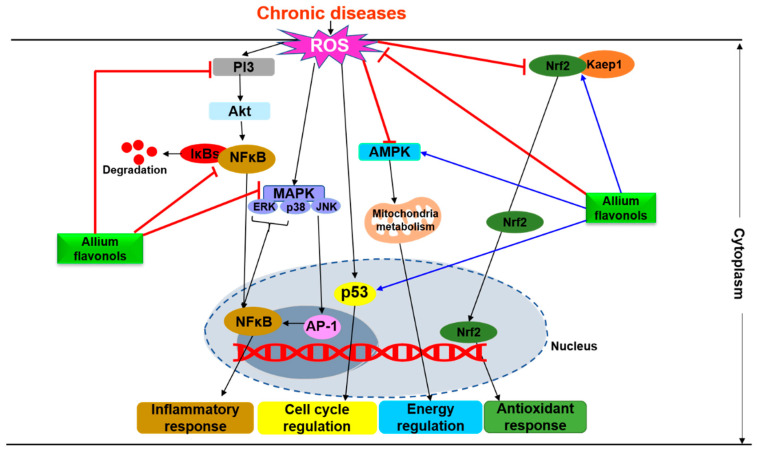
Examples of some molecular targets of *Allium*-derived flavonols during a chronic disease condition. Blue line indicates activation; red line indicates inhibiton; black line indicates pathways. AMPK, AMP-activated protein kinase; AP-1, Activator protein-1; ERK, extracellular signal-regulated kinase; JNK, jun N-terminal kinase MAPK, mitogen-activated protein kinase; PI3, phosphatidylinositol 3-kinase; Akt, protein kinase B; ROS, reactive oxygen species; NF-κB, nuclear factor kappa-light-chain-enhancer of activated B cells; Nrf2, nuclear factor erythroid 2-related factor 2.

Oxidative stress as denoted by the elevated levels of reactive oxygen species (ROS) has pleiotropic effects on the development of chronic diseases [137,138,139,140]. The inextricable correlations between the dietary intake of flavonols and their functional effects against certain chronic disorders are evident. Mechanistically, the health benefits of the *Allium* flavonols are mainly attributed to their modulatory effects on oxidative stress and inflammation (Table 3). During a chronic disease condition, these compounds can regulate the cellular ROS levels by various mechanisms involving direct scavenging and detoxification as well as engagement of redox signaling pathways (Figure 3). We present here a few examples of molecular targets of *Allium*-derived flavonols.

The transcription factor, nuclear factor erythroid 2-related factor 2 (Nrf2) is a master switch of cellular antioxidant response [138,141,142]. The active Nrf2 after its dissociation from its suppressor (Kelch-like ECH-associated protein 1 (Kaep1)) translocates from cytoplasm to the nucleus and controls the basal and inducible expression of the target genes that contain antioxidant response element (ARE) [143]. These target genes include antioxidant enzymes: Superoxide dismutase (SOD), catalase (CAT), glutathione reductase (GR), glutathione peroxidase (GPx), glutamate cysteine ligase (GCL), thioredoxin reductase (TrxR), peroxiredoxin (Prx), and sulfiredoxin (Srxn); detoxifying proteins: Heme oxygenase 1 (HO-1), glutathione *S*-transferase (GST) and NAD(P)H: Quinone oxidoreductase 1 (NQO1), and anti-inflammatory proteins: IL-6 and IL-1β [142]. Nrf2 also regulates inflammation through the (1) direct regulation of antioxidant enzymes and pro-inflammatory genes and (2) crosstalk with nuclear factor-κB (NF-κB) pathway [142,144]. In addition to regulating oxidative stress and inflammation, Nrf2 also coordinates different pathways involved in glucose and lipid metabolisms [145]. The mechanism of Nrf2 activation was concluded to be through multiple kinase pathways, including PI3K/Akt, MAPK, p38, glycogen synthase kinase (GSK), AMPK, and PKC [141,146,147]. Numerous studies have shown that flavonols and their glycosides confer protection against oxidative stress through the activation of Nrf2-ARE signaling [148,149,150,151,152,153]. For instance, quercetin alleviated oxidative stress by upregulating the Nrf2-ARE-mediated gene expression in vitro (NQO1) and in vivo (SOD, NQO1, and HO-1) through the MAPK (JNK, ERK, p38) signaling pathway [148,153]. Hussein et al. [152] reported that kaempferol exerted protective effects against oxidative stress by inducing Nrf2-ARE-mediated gene expression of SOD, CAT, and GPx through the modulation of GSK signaling pathway in rat models. Yang et al. [149] ascribed the antioxidant activity of isorhamnetin to the increased Nrf2 activity and its target gene expression of HO-1 and GCL via the phosphorylation of ERK1/2, PKCδ, and AMPK.

Nuclear factor kappa-B (NF-κB) is a family of transcription factors that plays a pivotal role in multiple aspects of innate and adaptive immune functions. Normally, NF-κB exists in the cytoplasm as an inactive complex in physical association with inhibitory proteins called inhibitors of κB (IκBs) [154]. Upon activation, NF-kB triggers inflammatory response by increasing the expression of pro-inflammatory cytokines (TNF-α, IL6, IL1-β), chemokines (monocyte chemoattractant protein-1, MCP-1), and adhesion molecules as well as by regulating the cell proliferation, apoptosis, morphogenesis, and differentiation [154,155]. The transcription of NF-κB-dependent genes also influences the levels of ROS and vice versa [156]. Therefore, a tightly regulated NF-κB signaling is essential to prevent any exacerbated inflammatory responses during a chronic illness [157]. Previous reports found that quercetin [158,159,160], myricetin [161], fisetin [162], and isorhamnetin [163] have suppressed the inflammatory responses via the inhibition of NF-κB signaling in animal models and human with chronic diseases. These studies suggest that the transcriptional specificity of NF-κB may be shaped by the crosstalk with other signaling pathways (p38, MAPK and ERK) and transcription factors (Nrf2 and p53). Furthermore, some enzymes including inducible nitric oxide synthase (iNOS), xanthine oxidase, cyclooxygenase-2 (COX-2), arachidonate 12-lipoxygenase, arachidonate 5-lipoxygenase, and cytochrome p450 enzymes that promote the production ROS are also regulated by NF-κB signaling [156].

p53, a transcription factor that regulates cell proliferation, senescence, DNA repair, and cell death and its activity is modulated by the degree of oxidative stress imposed [164]. The low levels of oxidative stress inhibit p53 expression, which promotes cell survival and repair by direct scavenging of free radicals through the expression of antioxidant genes [165]. Reciprocally, the acute/high stress promotes p53 expression and initiates DNA fragmentation to induce apoptosis via caspase cascade signaling [165]. Several flavonols are reported to cause cell cycle arrest at G2/M phase and triggered apoptosis in different types of human cancer cells via the activation of p53 [54,166,167].

Mitochondria-mediated ROS production accounts for approximately 90% of the total cellular ROS [168]. Lagoa et al. [169] identified mitochondrial respiratory chain complex-I and cyt c as the major molecular targets of quercetin and kaempferol in alleviating oxidative stress. Another mechanism pertaining to the beneficial effect of flavonols is identified as mitochondrial uncoupling, a regulated proton leak mediated by uncoupling proteins (UCPs) [170], which occurs through aldehydic lipid-peroxidation intermediates such as 4-hydroxy-2-nonenal [85] and AMPK activation [64]. The mitochondrial uncoupling activity of flavonols is also reported to induce apoptosis in cancer cells by dissipating MMP (Δψ_m_) through the activation of caspase cascades [54]. Influx of Ca^2+^ into the mitochondria is a critical for the availability of ATP since major enzymes in the tricarboxylic acid cycle are activated by Ca^2+^ [171]. Quercetin [87] and kaempferol [172] are reported to modulate mitochondrial Ca^2+^ accumulation and thereby reduce ROS emission.

Different protein kinase pathways including PKC [88], PI3K/Akt [54], MAPK [153], and AMPK [64,65] have been identified as the targets of allium derived flavonols for providing cell survival signaling. PI3K/Akt and MAPK (ERK, p38, and JNK) signaling pathways play critical roles in the regulation of cell proliferation, apoptosis, and autophagy [173] while AMPK is identified as a key regulator of energy homeostasis [174]. The AMPK activation is reported to be beneficial for both the prevention and treatment of a wide variety of metabolism related chronic diseases via its regulatory effects on fatty acid, cholesterol, carbohydrate, and amino acid metabolism as well as autophagy, mitochondrial function (biogenesis, fission and mitophagy) and cell growth [174]. Flavonols are reported to mediate cell cycle arrest at G2/M phage, apoptosis, and autophagy in different types of cancer cells via suppressing the expression of (PI3K)/Akt [54] and MAPKs [153] as well as increasing the expression of AMPK [64,65] signaling pathways.

Although the precise molecular details of the beneficial effects of *Allium*-derived flavonols not fully elucidated, these studies suggest that some cryptic mechanisms of action are subtly involved.

## 5. Bioavailability of *Allium*-Derived Flavonols

**Figure 4:**
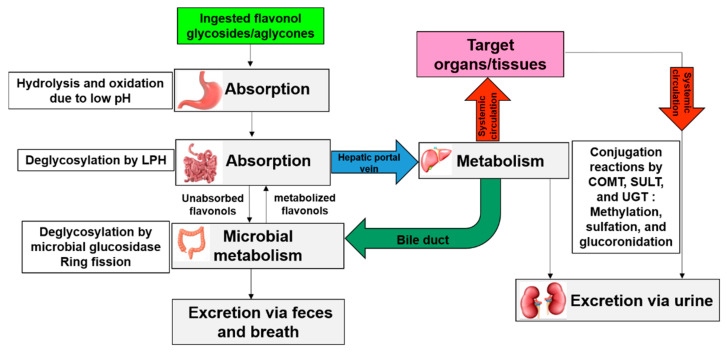
A simplified schematic representation of oral bioavailability of flavonols in humans. LPH, phlorizin lactase; COMT, catechol-*O*-methyl-transferases; SULT, sulfotransferases; UGT, uridine 5’-diphospho-glucuronosyl transferases.

Bioavailability refers to the amount of a compound/nutrient that enters systemic circulation and reaches the intended biological tissues [175,176]. Absorption, metabolism, and excretion are the main indices of bioavailability of an ingested compound [177,178]. The molecular actions of *Allium* flavonols are largely dependent on their bioavailability at the target tissue of humans. However, the bioavailability studies often involve the use of flavonoid-rich alliums rather than a pure isolated compound, where the possibility of the effects induced by other phytochemicals could not be excluded. Quercetin and its glucosides contribute to the major dietary flavonol intake in humans and have been the most extensively studied compounds for their bioavailability [177,179,180]. However, it should be noted that the complete bioavaialbility of quercetin in the body is not clearly understood. After quercetin, kaempferol contributes significantly to the flavonol intake in humans [181]. Nevertheless, there is a lack of scientific data on the bioavailability of *Allium*-derived kaempferol, isorhamnetin, myricetin, fisetin, and morin in humans. A simplified scheme of dynamics of flavonol/flavonol glycosides inside the body from oral intake to excretion is shown in Figure 4.

The site and manner in which flavonols are absorbed depend on their chemical structure [180,182,183]. Crespy et al. [183] reported gastric absorption of quercetin, but not its glycosides in a rat model, indicating a limited contribution of stomach in the bioavailabilty of flavonols. The pioneering study by Hollman et al. [184] indicated that 52% absorption of onion-derived quercetin glucosides whereas, 24% of quercetin aglycone given as a pure compound was absorbed in the small intestine of healthy ileostomy volunteers. The greater absorption of quercetin glucoside is attributed to its water-soluble nature and is mediated by sodium-dependent glucose transporter 1 (SGLT1) [184]. In contrast, higher quercetin aglycone (dried shallot skin) absorption is reported when quercetin aglycone was consumed as an integral food component as compared with its glucosides (shallot flesh) in humans [185]. This may be ascribed to the fact that quercetin aglycone is relatively lipophilic and thereby can easily enter intestinal enterocytes because of passive transport, whereas its respective glycosides must be hydrolyzed by phlorizin lactase or β-glucosidase located at the intestinal brush border membrane prior to absorption by an enterocyte [186,187]. Hollman et al. [188] pointed out that sugar moiety is the main determinant for the absorption of quercetin in humans; glucosides of quercetin being more efficiently absorbed than the rutinoside. However, the position of sugar moiety had no effects on their absorption when the same levels of pure 3-Qmg and 4’-Qmg were fed in humans [189]. Apart from glycosylation pattern, dietary source [188,190] and components of food matrix [191] also affect the oral bioavailability of flavonols. For example, higher absorption of quercetin from onions was reported as compared with apples in ileostomy patients [188] and healthy volunteers [190]. In an animal study using a porcine model, the fat content in the diet positively influences the bioavailability of 3-Qmg [191]. Dietary fat-dependent improvements in quercetin bioavailability, likely by enhancing its micellarization at the small intestine have also been reported in humans [192].

Once absorbed, all the flavonols are metabolized via three types of conjugation in the liver: Methylation, sulfation, and glucuronidation by catechol-*O*-methyl-transferases (COMTs) sulfotransferases (SULTs), and uridine 5’-diphospho-glucuronosyl transferases (UGTs), respectively, prior to reaching systemic circulation [177,186,193]. It is noteworthy to mention that only 5–10% of the total dietary polyphenols is absorbed in the small intestine, while the remaining 90–95% together with their conjugates (excreted through bile) reaches the colon and undergoes microbial metabolism, leading to the production of low molecular weight phenolic acids and aromatic compounds as well as CO_2_ [194,195,196,197]. The simpler phenolic compounds can be re-absorbed in the small intestine, then subjected to conjugation again in liver, reach the target tissues, and ultimately excreted via urine and feces as their respective conjugates, and via breath as CO_2_ [194,195,196,197].

The poor water solubility, low lipophilicity, and instability, as well as extensive first pass (intestine–liver) metabolism contribute to the low bioavailability of orally ingested flavonols [198]. Recently, research on developing novel delivery systems to improve their bioavailability, target-specificity and efficacy has gained much attention. In this context, various formulation strategies have been explored including preparations such as phospholipid complexes/phytosomes [199], liposomes [91], nanoparticles [200,201], nanoemulsions [202], solid dispersions [199,203], nanodispersions [204], nanocrystals [205], self-emulsifying systems [203], and prodrugs (structural modification) [206] as well as delivery as natural prodrugs [207].

## 6. Stability During Domestic and Technological Processing

Domestic and technological processing may bring a significant variability in allium flavonol contents. The kind and position of the sugar moiety in flavonol glycosides often determine their fate during these processing practices [208,209]. Data in the literature about the effects of the processing techniques on the flavonol content are restricted to onions only. For instance, Rodrigues et al. [210] reported that maceration of chopped red onion slightly reduced the levels of 3,4′-Qdg and 4′-Qmg during the first 5 h when kept at room temperature under continuous light exposure. However, Makris and Rossiter [211] observed no marked changes in 3,4′-Qdg and 4′-Qmg following 60 min of maceration of chopped onion, indicating the fact that longer time is needed for the enzyme action. Cooking significantly alters the flavonol content due to thermal degradation and transformation; however, changes vary with the type of culinary treatment and the length of exposure [209,210,211,212]. The losses of onion flavonols during different cooking treatments are supposedly affected in the following order: Boiling > microwave roasting > oven roasting > frying [210]. Intriguingly, Lee et al. [212] reported a very different trend of flavonol losses following various cooking treatments: Frying > sautéing > boiling in water with 3% salt > boiling in water with 1% salt > steaming > microwaving. However, baking resulted in the gain of onion flavonols. The high loss of flavonols after boiling and frying may be attributed to a combined effect of thermal degradation and leaching into cooking water and oil [209,210,212]. Thermal treatments led to deglycosylation of quercetin glucosides (4′-Qmg and 3,4′-Qdg) to their corresponding aglycone [209,210]. The sugar moiety attached at 3-*O*-position (C-ring) is more susceptible to thermal degradation as compared with that attached at 4-*O*-position (B-ring) [209]. Garlic and onion are often marketed as dried powders for culinary uses due to their longer shelf-life. Freeze-drying is considered as the best method to produce high-quality food powders with maximal retention of bioactive compounds in the final products as compared to other drying techniques [213]. Pérez-Gregorio et al. [214] reported that freeze-drying increased the flavonol levels (4′-Qmg and 3,4′-Qdg) in onions by 32% and no significant changes were observed in the flavonols of freeze-dried onions during 6-months of storage.

Fermentation of *Allium* vegetables is an important part of Asian cuisine. Several studies have substantiated the fermentation mediated changes of *Allium* flavonols. Most notably, the red onion fermentation by *Lactobacillus plantarum* S1 leads to the hydrolysis of quercetin diglucoside to its monoglucoside and aglycone derivatives [215]. Yang et al. [216] demonstrated that the controlled fermentation of yellow onion using a β-glucosidase-rich crude extract from *Aspergillus kawachii* resulted in elevated quercetin aglycone levels owing to the enzymatic cleavage of 4′-Qmg and 3,4′-Qdg. The regioselective de-glycosylation of onion quercetin glucosides by *Saccharomyces cerevisiae* is also reported following the fermentation [217]. Similarly, Lee et al. [218] demonstrated that onion fermentation using *Leuconostoc mesenteroides* at varying salt concentrations resulted in higher relative abundance of quercetin and isorhamnetin glucosides, while their corresponding aglycones were gradually increased via β-glucosidase action. Recently, Kothari et al. [40] also observed higher relative abundance of flavonol glycosides following the controlled *L. plantarum*-mediated fermentation of *A. tuberosum*. Contrastingly, no flavonol glucosides but only the traces of free quercetin are detected in onion following fermentation, suggesting a rapid decomposition of flavonols into smaller molecules including acetate, butyrate, and carbon dioxide [219]. Therefore, further studies should focus on a tailored microbial fermentation of alliums for the controlled production of bioactive metabolites toward enhancing the nutritional and health properties.

## 7. Food Fortification

The increasing nutrition knowledge has improved the consumer perception to opt for flavonoid fortified food products. Several researchers attempted to incorporate flavonol-rich alliums into food products to augment the health quality of foods. For instance, the onion skin extract addition improves the polyphenolic content and antioxidant activity of bread [220,221] and bean paste [222] in a dose-dependent manner. A cereal bar containing *A. fistulosum* extract (containing ferulic acid and quercetin) displayed anti-obesity effects in a rat model [223]. Recently, a quercetin derivative isolated from onion/beef soup demonstrated autophagy in the colon cancer cell in vitro [224]. However, not all the *Allium*-fortified foods have enhanced nutritional values. The fortification sometimes reported to negatively influence protein digestibility due to the formation of flavonoid–protein insoluble complexes [220,222]. Therefore, while designing an *Allium*-rich food formulation, the food matrix components also need to be considered to ensure its end-product quality. In addition, the scale-up and technical constraints related to the flavonol fortifications in food must be thoroughly evaluated considering their varying solubility, unstable nature, and low bioavailability in food matrices. Furthermore, biofortified products should also be subjected to public awareness, consumer participation, and acceptance parameters.

## 8. Future Perspectives and Limitations

From the above literature review, it is evident that the consumption of *Allium* flavonols may engender a lower risk of chronic disease development through modulating the oxidative stress and related low-grade systematic inflammation. However, the epidemiological studies regarding the health effects of *Allium* flavonols are surprisingly limited pertaining to several challenges including (1) time-consuming and labor-intensive isolation of a single bioactive flavonol from its natural source, (2) elusive underlying mechanism of action, and (3) their low bioavailability. Most of the *Allium*-derived flavonol studies are based on using solvent extracts that have multiple bioactive constituents such as OSCs, saponins, and other polyphenols and thus their additive or synergistic actions cannot be dismissed. In most of the studies reporting the health effects and related bioactivities, the specific flavonol compounds have not been fully characterized using ultra-high performance liquid chromatography (UHPLC) or gas chromatography (GC) coupled to high resolution-mass spectrometry (HR-MS), and nuclear magnetic resonance (NMR) spectroscopy. Hence, we emphasize on both functional and chemical characterization of the flavonols from *Allium* species having substantiated pharmacological effects. In addition, most of the mechanistic evidence derived from culture-based and/or animal model studies using the native forms of flavonol either in isolation or combination. However, following the ingestion, flavonols undergo a cascade of biotransformation reactions depending on individual’s genetics, dietary habits, and various environmental factors. The in vivo studies often use animal models of similar genetic makeup and involves normalization of diet as well as the environment. However, the humans are genetically diverse and exposed to numerous exogenous factors, and their diets often includes highly diverse nutritional components [225]. These variables make it very difficult to ascertain the true effect(s) of dietary *Allium*-derived flavonols on the human host. Therefore, mechanistic studies should be thrived in both animal models and humans with the help of integrated multi-omics approach. To address the low bioavailability issue, further research is needed toward developing micro- and nano-delivery systems to maximize the absorption of flavonols and thereby enhance their target specificity and therapeutic efficacy.

## 9. Conclusions

Long-term oxidative stress contributes to the development of several chronic conditions including age-related neurodegenerative and cardiovascular diseases, cancer, diabetes, obesity, and low-grade systematic inflammations, among many others. Disease prevention models practicing healthy lifestyle changes including dietary modifications are suggested to be one of the effective strategies to address these chronic disorders. Herein, we exclusively discuss the *Allium*-derived flavonols as natural antioxidants and their possible role toward the prevention of chronic diseases in humans through alleviating the oxidative stress and associated chronic inflammation. Nonetheless, the potential importance of other biologically active compounds, such as phenolic acids, anthocyanins, saponins, and OSCs of alliums, cannot be undermined. Currently, insufficient scientific evidence prevails to draw any conclusion on flavonol intake from alliums and their exclusive health benefits. Further mechanistic studies involving animal models and human volunteers are required to substantiate any potential health benefit claims. The poor bioavailability of flavonols and food matrix constituents should also be taken into consideration while designing any formulations. Finally, individual health conditions that could interfere the metabolism and, thus, the health effect should also be considered for the clinical use of *Allium*-derived flavonols.

## Figures and Tables

**Table 1:** Flavonol contents of some common edible *Allium* species.

Common Name	Scientific Name	Plant Part	Total Flavonol Content	References
Red Onion 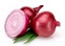	*A. cepa*	Bulb	415-1917 mg/kg F.W.	[22]
Yellow onion 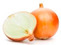	*A. cepa*	Bulb	270-1187 mg/kg F.W.	[22]
White onion 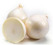	*A. cepa*	Bulb	7 mg/kg F.W.	[23]
Italian shallot 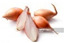	*A. ascalonicum*	Bulb	1023 mg/kg F.W.	[23]
French shallot 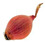	*A. ascalonicum*	Bulb	1167 mg/kg F.W.	[23]
Leek 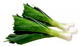	*A. porrum*	Bulb	246 mg/kg F.W.	[24]
Garlic 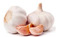	*A. sativum*	Cloves	16.19 mg/kg D.W.	[25]
Ramson bear’s garlic 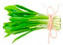	*A. ursinum*	Green leaves	1856.31 mg/100 g D.W.	[26]
		Yellow leaves	2362.96 mg/100 D.W.	
		Stalks	206.07 mg/100 g D.W.	
		Seeds	73.14 mg/100 g D.W.	
Ramps 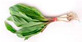	*A. tricoccum*	Leaves	11.81 mg/g D.W.	[27]
		Stem	0.0382 mg/g D.W.	
		Bulb	--	
Chinese chives 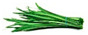	*A. odorum* (*A. tuberosum*)	Leaves	160 mg/kg D.W.	[28]
Welsh onion 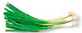	*A. fistulosum*	Leaves	2329 mg/kg D.W.	[28]
Yellow flowered garlic 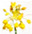	*A. flavum* subsp. *flavum*	Aerial parts	44-264 mg/g D.W.	[29]
		Bulb	0.77-832 µg/g D.W.	
Keeled garlic 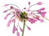	*A. carinatum*	Whole plant	11.14 mg/g D.W.	[30]

F.W.: Fresh weight; D.W.: Dry weight.

**Table 2:** Different kinds of flavonols and their glycosides identified in *Allium* species.

S. No.	Flavonol Aglycones/Glycosides	Plant Species	References
1	Quercetin (Que)	*A. cepa*	[22]
2	Que-3-*O*-glucoside	*A. cepa*, *A. sativum*, *A. flavum*, *A. macrostemon*	[16,22,29,37,38]
3	Que-4′-*O*-glucoside	*A. cepa*	[16]
4	Que-3,4′-*O*-diglucoside	*A. cepa*, *A. tuberosum*	[16,22,36]
5	Que-3-*O*-rutinoside	*A. cepa*, *A. chinense*	[22,39]
6	Que-7-*O*-glucoside	*A. cepa*	[22]
7	Que-7-*O*-rhamnoside	*A. cepa*	[22]
8	Que-7,4′-*O*-diglucoside	*A. cepa*	[22]
9	Que-3,7-*O*-diglucoside	*A. cepa*	[22]
10	Que-3,7,4′-*O*-triglucoside	*A. cepa*	[16,22]
11	Que-3-*O*-rhamnoside	*A. cepa*, *A. fistulosum*	[22]
12	Que dimer	*A. cepa*	[22]
13	4′-Glucoside of que dimer	*A. cepa*	[22]
14	Que trimer	*A. cepa*	[22]
15	Quercetin sophoroside glucuronide	*A. tricoccum*	[27]
16	Que hexoside glucuronide	*A. tricoccum*	[27]
17	Que sophoroside	*A. tuberosum*	[40]
18	Que-3-*O*-β-d-xylopyranoside	*A. sativum*	[37]
19	Kaempferol (Kae)	*A. cepa, A. tuberosum*	[36,41,42]
20	Kae-3-*O*-glucoside	*A. cepa*, *A. sativum*, *A. flavum*, *A. ursinum*, *A. macrostemon*	[22,29,37,38,43]
21	Kae-4′-*O*-glucoside	*A. cepa*	[22]
22	Kae-7,4′-*O*-diglucoside	*A. cepa*	[22]
23	Kae-7-*O*-glucoside	*A. triquetrum*	[44]
24	Kae-3,4′-*O*-diglucoside	*A. cepa*, *A. tuberosum*, *A. macrostemon*	[22,36,38]
25	Kae-3,7-di-*O*-rhamnoside	*A. roseum*	[45]
26	Kae-3,7-di-*O*-glucoside	*A. macrostemon*	[38]
27	Kae-3-*O-*glucuronide-7-*O*-rhamnosylglucoside	*A. roseum*	[46]
28	Kae-3-*O*-rutinoside	*A. roseum*, *A. tuberosum*, *A. triquetrum*	[36,44,46]
29	Kae-3-*O*-glucoside-7-*O*- glucuronide	*A. roseum*	[46]
30	Kae-7-*O*-glucuronide	*A. roseum*	[46]
31	Kae-3-*O*-glucuronide	*A. roseum*	[46]
32	Kae-7-*O*-(6”-malonyl)-glucoside	*A. roseum*	[46]
33	Kae-3-*O*-sophoroside	*A. tuberosum*, *A. tricoccum*	[27,36]
34	Kae-3-*O*-β-d-glucosyl-(1 2)-*O*-α-L-xylopyranoside	*A. tuberosum*	[36]
35	3-*O*-β-d-(2-*O*-feruloyl)-glucosyl-7,4’-di-*O*-β-d-glucosylkaempferol	*A. tuberosum*	[36]
36	3-*O*-β-sophorosyl-7-*O*-β-d-(2-*O*-feruloyl)-glucosylkaempferol	*A. tuberosum*	[36]
37	Kae-3-*O*-neohesperidoside	*A. ursinum*	[43]
38	Kae-3-*O*-flneohesperidoside-7-*O*-[2-*O*-(trans-p-coumaroyl)]-fl-d-glucopyranoside,	*A. ursinum*	[43]
39	Kae-3-*O*-fl-neohesperidoside-7-*O*-[2-*O*-(trans-feruloyl)]-fl-d-glucopyranoside	*A. ursinum*	[43]
40	Kae-3-*O*-fl-neohesperidoside-7-*O*-[2-*O*-(trans-p-coumaroyl)-3-*O*-flD-glucopyranosyl-1-fl-d-glucopyranoside	*A. ursinum*	[43]
41	Kae-3-*O*-[2-*O*-(trans-*p*-coumaryl)-β-d-galactopyranosyl]-(1→4)-*O*-β-d-glucopyranoside	*A. porrum*	[47]
42	Kae-3-O-[2-*O*-(trans-p-coumaryl)-β-d-glucopyranosyl]-(1→6)-*O*-β-d-glucopyranoside	*A. porrum*,*A. triquetrum*	[44,47]
43	Kae-3-*O*-(2-*O*-trans-p-feruloyl)glucoside	*A. triquetrum*	[44]
44	8-hydroxykaempferol 8-*O*-glucoside	*A. triquetrum*	[44]
45	Kae-3-*O*-[2-*O*-(trans-p-coumaroyl)-3-*O*-β-d-glucopyranosyl]-β-d-glucopyranoside	*A. triquetrum*	[44]
46	Isorhamnetin (Iso)	*A. cepa*	[48]
47	Iso-4′-*O*-glucoside	*A. cepa*	[16,22]
48	Iso-3-*O*-glucoside	*A. cepa*, *A. vineale*, *A. macrostemon*	[22,38,49]
49	Iso-3,4′-*O*-diglucoside	*A. cepa*; *A. tuberosum*	[16,22,40]
50	Iso-4′-*O*-galactoside	*A. cepa*	[16]
51	Myricetin	*A. cepa*	[41]
52	Fisetin	*A. cepa*	[41]
53	Morin	*A. cepa*	[50]

Que: Quercetin; Kae: Kaempferol; Iso: Isorhamnetin.

**Table 3:** *Allium* derived flavonols for chronic disease prevention in pe-clinical and clinical studies.

Plant Species (Part Used)	Major Identified Flavonol(s)	Study System, Dose, and Duration	Activity	Biological Effects	Molecular Targets	References
*A. cepa* (peel)	Quercetin, quercitrin, kaempferol, and morin	In vitro: HT–29, 50–250 µg/mL for 24 h	Anticancer	- Inhibits proliferation - Reduces oxidative stress - Reduces inflammation	↑ LDH release; ↓ HO-1; ↓ TNF-α; ↓ GSTs, GSTM1, GSTT1, and GSTP1	[50]
*A. cepa* (bulb)	Quercetin 3,7,4′-triglucoside, quercetin 7,4′- diglucoside, quercetin 3,4′-diglucoside, isorhamnetin 3,4′-diglucoside, quercetin 3-glucoside, quercetin 4′-glucoside, isorhamnetin 4′-galactoside, and isorhamnetin 4′-glucoside	In vitro: THP-1, K562, and U937, 20–100 µg/mL for 48 h	Anticancer	- Inhibits cell proliferation - Induces apoptosis	↓ Caspase-3, -8 and -9 activity; ↑ Bid; ↓ Bcl-xL; ↑ DR5 TRAIL; ↓ Survivin; ↓ cIAP-1; ↓ PI3K/Akt	[16]
*A. cepa* (bulb)	-do-	In vitro: AGS, 1–100 µg/mL for 48 h	Anticancer	- Inhibits cell proliferation - Induces apoptosis	↑ PAPR; ↓ Procaspase-3; ↓ Bcl-2; ↑ Bid; ↑ Bax; ↑ p53; ↓ MMP (Δ Ψm); ↓ PI3K/Akt	[54]
*A. cepa* (scale)	Quercetin and quercetin-4′-β-*O*-d-glucoside	In vivo: Atypical prostatic hyperplasia model of Wistar rats, 75, 150, or 300 mg/kg/d orally for 30 d	Anticancer	- Inhibits proliferation - Induces apoptosis - Reduces inflammation	↓ IL-6; ↓ IL-8; ↓ TNF-α; ↓ IGF-1	[14]
*A. cepa* (solid waste)	Quercetin, quercetin-3,4′-*O*-diglucoside, and quercetin-4′-*O*-monoglucoside	In vitro: ACHN, Panc 1, Calu 1, H460, and HCT 116, 1–5 mg/mL for 24 h	Anticancer	- Inhibits proliferation	n.r.	[67]
*A. cepa* var*. proliferum* (stems)	Isorhamnetin and kaempferol	In vitro: HepG2, 20–100 mg/mL for 72 h	Anticancer	- Inhibits proliferation - Induces apoptosis	n.r.	[68]
*A. cepa* (n.r.)	Rutin	In vivo: Hyperlipidemia colon tumor model of BALB/C nu/nu mice, 100-300 mg/kg/d intragastrically for 3 wk	Antihyperlipidemic and anticancer	- Improves lipid metabolism - Inhibits tumor proliferation	n.r.	[69]
*A. cepa* (peel)	Quercetin	In vivo: HFD-fed SD rats, 0.2% in diet for 8 wk	Antiobesity	- Reduces mesenteric fat	↑ Adiponectin; ↓ PPAR-γ	[61]
*A. cepa* (peel)	Quercetin	In vitro: 3T3-L1, 25–100 µg/mL for 24 h In vivo: HFD-fed SD rats, 0.36% or 0.72% in diet for 8 wk	Antiobesity	- Attenuates lipid metabolism - Reduces body weight - Reduces adipose tissue - Improves lipid metabolism	↓ AP-2; ↑ CPT-1α; ↑ FABP4; ↓ PPAR-γ ↓ C/EBP-α; ↓ FAS; ↓ ACC ↑ CPT-1α; ↑ UCP-1	[62]
*A. cepa* (peel)	Quercetin	In vitro: 3T3-L1, 1–4 μg/mL for 24 h	Antiobesity	- Reduces lipid accumulation - Reduces adipogenesis - Induces lipolysis	↓ GPDH activity; ↓ PPAR-γ; ↓ C/EBP-α ↓ AP2; ↓ LPL; ↑ ATGL; ↑ HSL	[63]
*A. cepa* (peel)	Quercetin and isoquercetin	In vitro: 3T3-L1, 50–150 μg/mL for 11 d (on day 5, 7, and 9) In vivo: HFD-fed C57BL/6 mice, 0.5% in diet for 8 wk	Antiobesity	- Induces adipocyte browning - Reduces adipogenesis - Reduces lipogensis	↓ PPAR-γ; ↓ ACC; ↓ FAS; ↑ PRDM16; ↑ UCP1; ↓ FGF21; ↑ TBX1; ↓ CIDEA; ↑ PGC1α; ↑ CPT1-α ↓ ACC; ↑ PRDM16; ↑ UCP1; ↑ FGF21; ↑ CIDEA; ↑ PGC1α	[64]
*A. cepa* (peel)	Quercetin	Randomized, double-blind, placebo-controlled study: Obese women, 100 mg/d (50 mg bis in die) orally for 12 wk	Antiobesity	- Reduces waist and hip circumferences - Reduces oxidative stress	↓ ROS; ↑ SOD activity	[70]
*A. fistulosum* (bulbs and roots)	Quercetin	In vivo: HFD-obese C57BL/6 J mice, 100 mg/kg/d orally for 6 wk	Antiobesity	- Reduces body weight - Improves lipid and glucose metabolism	↑ AMPK (AMPKα1 and AMPKα2); ↑ Adiponectin; ↑ UCP2; ↓ PPAR-γ	[65]
*A. cepa* (peel)	Quercetin (Q) and quercetin monoglucoside (Qmg)	In vivo: HFD-fed Wistar rats, 0.21% (Q) or 0.36% (Q+Qmg) in diet for 4 wk	Antiobesity	- Reduces oxidative stress - Improves lipid metabolism - Increases gut microbial enzyme activity	n.r.	[71]
*A. chinense* (bulbs)	Quercetin and rutin	In vivo: HFD-fed Wistar rats, 0.09 or 0.18% per day orally for 12 wk	Anti-hyperlipidemic	- Improves lipid metabolism	n.r.	[39]
*A. cepa* (skin)	Quercetin	In vivo: OGTT in SD rats, 0–500 mg/kg, single oral dose	Antidiabetic	- Reduces post-prandial blood glucose - Inhibits carbohydrate hydrolases (sucrase and maltase)	n.r.	[72]
*A. cepa* (peel)	Quercetin	In vivo: HFD/STZ-diabetic SD rats, 0.5 or 1% in diet for 8 wk	Antidiabetic and antioxidant	- Increases IAUC - Reduces blood glucose - Reduces fasting blood glucose - Increases glycogen levels - Reduces oxidative stress - Reduces inflammation	↑ INSR and GLUT4 ↑ SOD activity; ↓ MDA level; ↓ IL-6	[73]
*A. cepa* (bulb)	Kaempferol-3-*O*-β-d-6{*p*- coumaroyl} glucopyranoside	In vivo: Alloxan-diabetic Wistar rats, 25 mg/kg single oral dose	Antidiabetic	- Reduces blood glucose	n.r.	[74]
*A. cepa* (n.r.)	Quercetin and quercetin glycosides	In vitro: *Xenopus laevis* oocytes, 1–15 mg/mL for 30 min In vivo: OGTT in HFD fed C57BL/6N mice, 14 mg single oral dose	Antidiabetic	- Reduces glucose uptake	↓ SGLT1	[75]
*A. tuberosum* (leaves)	Kaempferol glycoside derivatives	In vivo: Alloxan-diabetic Wistar rats, 100-400 mg/kg/d orally for 30 d	Antidiabetic	- Reduces oxidative stress - Reduces fasting blood glucose - Improves lipid metabolism	↑ GSH; ↑ SOD and CAT activities	[36]
*A. tuberosum* (leaves)	Kaempferol glycoside	In vivo: HFD/STZ-diabetic Wistar rats, 100 or 400 mg/kg/d orally for 40 d	Antidiabetic	- Reduces renal oxidative stress - Reduces inflammation - Reduces blood glucose - Improves renal and serum lipid profiles - Reduces serum creatinine - Reduces blood urea nitrogen - Reduces urinary albumin levels	↑ GSH; ↑ CAT and SOD activities; ↓ TGF-β1; ↓ TNF-α; ↓ IL-6; ↓ IL-1β	[76]
*A. cepa* (peel)	Quercetin	Randomized, double-blind, placebo-controlled parallel design: Healthy smokers, 100 mg/d for 10 wk	Cardioprotective	- Lowers blood pressure - Improves lipid profiles - Lowers blood glucose	⇔ Inflammatory markers	[77]
*A. cepa* (outer skin)	Quercetin, quercetin 4′-glucoside, and quercetin 3,4′-diglucoside	In vivo: HFD-fed Wistar rats, 5% in diet for 18 wk	Cardioprotective	- Atherogenic index - Lowers incremental elastic modulus	n.r.	[78]
*A. cepa* (peel)	Quercetin	In vitro: HUVEC, 50 and 100 μg/mL for 1 h In vivo: SD rats, 2 or 10 mg/d orally for 6 wk	Cardioprotective	- Delays arterial thrombus formation	↓ Tissue factor; ↓ JNK and ERK (MAPK)	[79]
*A. cepa* (peel)	Quercetin	Epidemiologic study: Healthy men, 4.3 g/d orally for 30 d	Cardioprotective	- Improves postprandial flow-mediated dilation	n.r.	[80]
*A. cepa* (peel)	Quercetin	Randomized double-blind, placebo-controlled prospective trial: Healthy overweight and obese individuals, 100 mg/d (50 mg twice daily) orally for 12 wk	Cardioprotective	- Improves flow-mediated dilation - Improves circulating endothelial progenitor cell count	n.r.	[81]
*A. cepa*(peel)	Quercetin quercetin hexoside 1, quercetin hexoside 2, quercetin dihexoside, methylquercetin hexoside, kaempferol, and methyl quercetin	Randomized double-blind, placebo-controlled prospective trial: Overweight-to-obese patients with (pre-)hypertension, 162 mg/d for 6 wk	Cardioprotective	- Lowers systolic ambulatory blood pressure	⇔ Biomarkers of inflammation and endothelial function	[82]
*A. cepa* (peel)	Quercetin	In vitro: SD rat platelets, 50–500 μg/mL for 3 min	Cardioprotective	- Inhibits platelet aggregation - Reduces oxidative stress	↓ TXA2 production; ↓ TXAS and COX-1 activity; ↓ Intracellular Ca^2+^; ↑ cAMP	[83]
*A. cepa* (tunic)	Quercetin, quercitrin, isoquercitrin, rutin, and kaempferol	In vivo: Wistar rats, 10 mg/kg/d, orally for 14 d	Cardioprotective	- Lowers blood pressure parameters - Reduces oxidative stress	↑ SOD and CAT activity; ↑ GSH levels	[42]
*A. cepa* (bulb)	Quercetin, quercetin-3,4′-*O*-diglucoside, and quercetin-4′-*O*-monoglucoside	In vitro: SD Rat platelet-rich plasma, 1–5 mg/mL for 5 min	Cardioprotective	- Inhibits platelet aggregation	n.r.	[84]
*A. flavum* and *A. carinatum* (whole plant)	Quercetin, kaempferol, isorhamnetin, rutin, quercetin 3-*O*-glucoside, and kaempferol-3-*O*-glucoside,	In vitro: A549 and HepG215-125 µg/mL for 24 h In vivo: Doxorubicin-induced toxicity in zebrafish embryos, 1–60 μg/mL for 96 h	AnticancerCardioprotective and myeloprotective	- Reduces oxidative stress - Reduces cardiovascular and morphological abnormalities - Anti-angiogenesis	↑ SOD and CAT activity	[30]
*A. cepa* (bulb)	Quercetin	In vivo: Ischemia/reperfusion induced injury in gerbil hippocampus, 50 or 100 mg/kg/d orally for 15 d	Neuroprotection	- Reduces lipid peroxidation - Attenuates activations of astrocytes and microglia	↓ 4-hydroxy-2-nonenal	[85]
*A. victorialis* (leaves)	Kaempferol and quercetin glycosides	In vitro: LPS-activated BV-2 cells, 20 μM for 24 h	Neuroprotection	- Anti-inflammatory effects	↓ NO production	[86]
*A. cepa* (bulb)	Quercetin	In vitro: glutamate-mediated oxidative stress in HT22 cells, 1–25 µM for 12 h	Neuroprotection	- Reduces apoptosis	↓ ROS; ↓ Ca^2+^ influx; ↑ MMP (ΔΨ_m_); ↓ Bid and Bax ↓ MAPKs (ERK, JNK, and p38)	[87]
*A. cepa* (bulb)	Quercetin	In vitro: BSO-induced oxidative stress in mouse neocortices, 1–10 mg/mL, for 30 min	Neuroprotection	- Reduces oxidative stress	↓ ROS; ↓ LDH release; ↑ ERK1/2; ↓ p38MAPK; ↓ PKC-ε	[88]
*A. cepa* (bulb)	Quercetin	In vivo: AlCl_3_ induced injury in Swiss albino mice, 50, 100 or 200 mg/kg/d orally for 60 d	Neuroprotection	- Improves muscle coordination and memory deficits - Reduces oxidative stress - Reduces inflammation	Acts as PPARγ agonist ↓ ROS; ↑ GSH, CAT ↓ AChE	[89]
*A. cepa* (bulb)	Quercetin 3,4′-*O*-β-d-diglucoside	In vitro: HSP cells under nutrient deprived condition, 0.1–500 µM for 20 h	Neuroprotection	- Reduces apoptosis - Alters cell morphology	↑ Ki-67; ↓ Bax/Bcl-2; ↑ Adhesion molecules (pan-cadherin and focal adhesion kinase)	[90]
*A. cepa* (outer scale)	Quercetin	In vivo: Cerebral ischemia/reperfusion-induced injury in Swiss Albino mice, 85 mg/kg/d for 7 d	Neuroprotection	- Improves cognitive/sensorimotor functions - Reduces cerebral infarct size - Reduces brain oxidative stress	↑ GSH; ↑ SOD activity ↑ TBARS	[91]

↓: downregulated; ↑: upregulated; ⇔: no effect; n.r.: not reported. ACC, Acetyl-CoA carboxylase; AChE, acetylcholinesterase; AFABP, adipocyte fatty acid-binding protein; Akt, protein kinase B; AlCl_3_, aluminium chloride; AMPK, AMP-activated protein kinase; AP, activator protein; ATGL, adipose triglyceride lipase; Bax, B-cell lymphoma 2 associated X protein; Bcl-2, B-cell lymphoma; Bcl-xL, B-cell lymphoma extra large; Bid, BH3-interacting domain death agonist; BSO, L-buthionine-S, R-sulfoximine; cAMP, cyclic adenosine monophosphate; CAT, catalase; C/EBPα, CCAAT-enhancer-binding protein homologous protein alpha; cIAP-1, cellular inhibitor of apoptosis protein-1; CIDEA, cell death-inducing DNA fragmentation factor-alpha-like effector A; COX, cyclooxygenase; CPT-1α, carnitine palmitoyltransferase-1α; DR, death receptor; ERK 1/2, extracellular-signal-regulated kinase 1/2; FABP4, fatty acid binding protein 4; FAS, Fas cell surface death receptor; FGF, fibroblast growth factor; GLUT4, glucose transporter type 4; GPDH, glycerol-3-phosphate dehydrogenase; GSH, glutathione; GST, glutathione *S*-transferase; HFD, high-fat diet; HO-1, heme oxygenase-1; HSL, hormone-sensitive lipase; INSR, insulin receptor; IGF-1, insulin-like growth factor 1; IL, interleukin; JNK, Jun N-terminal kinase; Ki-67, nuclear protein; LDH, lactate dehydrogenase; LPL, lipoprotein lipase; LPS, lipopolysaccharide; MAPK, mitogen-activated protein kinase; MDA, malondialdehyde; MMP, mitochondrial membrane potential; NF-κB, nuclear factor kappa B; NO, nitric oxide; Nrf2, nuclear related factor 2; OGTT, oral glucose tolerance test; PARP-γ, peroxisome proliferator-activated receptor-gamma; PGC1α, PARP-γ coactivator 1-alpha; PI3K, phosphatidylinositol 3-kinase; PKC-ε, protein kinase C; PRDM, positive regulatory domain; 3-Qmg, quercetin-3-*O*-glucoside; 4′-Qmg, quercetin-4′-*O*-glucoside; 3,4′-Qdg, quercetin-3,4′-di-*O*-glucoside; ROS, reactive oxygen species; SGLT1, sodium-glucose linked transporter 1; SOD, superoxide dismutase; STZ, streptozotocin; TBARS, thiobarbituric acid reactive substances; TBX1, T-box 1; TGF-β1, transforming growth factor beta 1; TNF-α, tumor necrosis factor alpha; TRAIL, TNF-related apoptosis-inducing ligand; TXA2, thromboxane A2; TXAS, TXA2 synthase; UCP, uncoupling protein.

## References

[1] World Health Organization (2018). World Health Statistics 2018: Monitoring Health for the SDGs Sustainable Development Goals.

[2] Miller V., Webb P., Micha R., Mozaffarian D., Database G.D. (2020). Defining diet quality: A synthesis of dietary quality metrics and their validity for the double burden of malnutrition. Lancet Planet. Health.

[3] Bousquet J., Anto J.M., Iaccarino G., Czarlewski W., Haahtela T., Anto A., Akdis C.A., Blain H., Canonica G.W., Cardona V. (2020). Is diet partly responsible for differences in COVID-19 death rates between and within countries?. Clin. Transl. Allergy.

[4] Gupta R., Hussain A., Misra A. (2020). Diabetes and COVID-19: Evidence, current status and unanswered research questions. Eur. J. Clin. Nutr..

[5] He W., Chen L., Chen L., Yuan G., Fang Y., Chen W., Wu D., Liang B., Lu X., Ma Y. (2020). COVID-19 in persons with haematological cancers. Leukemia.

[6] Butler M.J., Barrientos R.M. (2020). The impact of nutrition on COVID-19 susceptibility and long-term consequences. Brain Behav. Immun..

[7] Muscogiuri G., Barrea L., Savastano S., Colao A. (2020). Nutritional recommendations for CoVID-19 quarantine. Eur. J. Clin. Nutr..

[8] Chandra R.K. (1992). Effect of vitamin and trace-element supplementation on immune responses and infection in elderly subjects. Lancet-Lond.

[9] Teshika J.D., Zakariyyah A.M., Zaynab T., Zengin G., Rengasamy K.R., Pandian S.K., Fawzi M.M. (2019). Traditional and modern uses of onion bulb (*Allium cepa* L.): A systematic review. Crit. Rev. Food Sci. Nutr..

[10] Putnik P., Gabrić D., Roohinejad S., Barba F.J., Granato D., Mallikarjunan K., Lorenzo J.M., Kovačević D.B. (2019). An overview of organosulfur compounds from *Allium* spp.: From processing and preservation to evaluation of their bioavailability, antimicrobial, and anti-inflammatory properties. Food Chem..

[11] Hansen E.A., Folts J.D., Goldman I.L. (2012). Steam-cooking rapidly destroys and reverses onion-induced antiplatelet activity. Nutr. J..

[12] Kyung K.H. (2012). Antimicrobial properties of allium species. Curr. Opin. Biotechnol..

[13] Poojary M.M., Putnik P., Kovačević D.B., Barba F.J., Lorenzo J.M., Dias D.A., Shpigelman A. (2017). Stability and extraction of bioactive sulfur compounds from *Allium* genus processed by traditional and innovative technologies. J. Food Compos. Anal..

[14] Elberry A.A., Mufti S., Al-Maghrabi J., Abdel Sattar E., Ghareib S.A., Mosli H.A., Gabr S.A. (2014). Immunomodulatory effect of red onion (*Allium cepa* Linn) scale extract on experimentally induced atypical prostatic hyperplasia in Wistar rats. Mediat. Inflamm..

[15] Lee H.-A., Han S.-J., Hong S., Kim D.-W., Oh G.-W., Kim O. (2014). Onion peel water extracts enhance immune status in forced swimming rat model. Lab. Anim. Res..

[16] Han M.H., Lee W.S., Jung J.H., Jeong J.-H., Park C., Kim H.J., Kim G., Jung J.-M., Kwon T.K., Kim G.-Y. (2013). Polyphenols isolated from *Allium cepa* L. induces apoptosis by suppressing IAP-1 through inhibiting PI3K/Akt signaling pathways in human leukemic cells. Food Chem. Toxicol..

[17] Neveu V., Perez-Jiménez J., Vos F., Crespy V., du Chaffaut L., Mennen L., Knox C., Eisner R., Cruz J., Wishart D. (2010). Phenol-Explorer: An online comprehensive database on polyphenol contents in foods. Database.

[18] Rauter A.P., Ennis M., Hellwich K.-H., Herold B.J., Horton D., Moss G.P., Schomburg I. (2018). Nomenclature of flavonoids (IUPAC Recommendations 2017). Pure Appl. Chem..

[19] Ververidis F., Trantas E., Douglas C., Vollmer G., Kretzschmar G., Panopoulos N. (2007). Biotechnology of flavonoids and other phenylpropanoid-derived natural products. Part I: Chemical diversity, impacts on plant biology and human health. Biotechnol. J. Healthc. Nutr. Technol..

[20] Pérez-Gregorio R.M., García-Falcón M.S., Simal-Gándara J., Rodrigues A.S., Almeida D.P. (2010). Identification and quantification of flavonoids in traditional cultivars of red and white onions at harvest. J. Food Compos. Anal..

[21] Robards K., Antolovich M. (1997). Analytical chemistry of fruit bioflavonoids: A review. Analyst.

[22] Slimestad R., Fossen T., Vågen I.M. (2007). Onions: A source of unique dietary flavonoids. J. Agric. Food Chem..

[23] Bonaccorsi P., Caristi C., Gargiulli C., Leuzzi U. (2008). Flavonol glucosides in *Allium* species: A comparative study by means of HPLC–DAD–ESI-MS–MS. Food Chem..

[24] Soininen T.H., Jukarainen N., Auriola S.O., Julkunen-Tiitto R., Karjalainen R., Vepsäläinen J.J. (2014). Quantitative metabolite profiling of edible onion species by NMR and HPLC–MS. Food Chem..

[25] Kim J.-S., Kang O.-J., Gweon O.-C. (2013). Comparison of phenolic acids and flavonoids in black garlic at different thermal processing steps. J. Funct. Foods.

[26] Oszmianski J., Kolniak-Ostek J., Wojdyło A. (2013). Characterization and content of flavonol derivatives of *Allium ursinum* L. plant. J. Agric. Food Chem..

[27] Dabeek W.M., Kovinich N., Walsh C., Ventura Marra M. (2019). Characterization and Quantification of Major Flavonol Glycosides in Ramps (*Allium tricoccum*). Molecules.

[28] Miean K.H., Mohamed S. (2001). Flavonoid (myricetin, quercetin, kaempferol, luteolin, and apigenin) content of edible tropical plants. J. Agric. Food Chem..

[29] Simin N., Orcic D., Cetojevic-Simin D., Mimica-Dukic N., Anackov G., Beara I., Mitic-Culafic D., Bozin B. (2013). Phenolic profile, antioxidant, anti-inflammatory and cytotoxic activities of small yellow onion (*Allium flavum* L. subsp. *flavum*, Alliaceae). LWT-Food Sci. Technol..

[30] Aleksandar P., Dragana M.-Ć., Nebojša J., Biljana N., Nataša S., Branka V., Jelena K.-V. (2019). Wild edible onions—*Allium flavum* and *Allium carinatum*—successfully prevent adverse effects of chemotherapeutic drug doxorubicin. Biomed. Pharmacother..

[31] Zheng Y.-Z., Deng G., Liang Q., Chen D.-F., Guo R., Lai R.-C. (2017). Antioxidant activity of quercetin and its glucosides from propolis: A theoretical study. Sci. Rep..

[32] Rice-Evans C.A., Miller N.J., Paganga G. (1996). Structure-antioxidant activity relationships of flavonoids and phenolic acids. Free Radic. Biol. Med..

[33] Rodrigues A.S., Pérez-Gregorio M.R., García-Falcón M.S., Simal-Gándara J., Almeida D.P. (2010). Effect of post-harvest practices on flavonoid content of red and white onion cultivars. Food Control.

[34] Rodrigues A.S., Pérez-Gregorio M.R., García-Falcón M.S., Simal-Gándara J., Almeida D.P.F. (2011). Effect of meteorological conditions on antioxidant flavonoids in Portuguese cultivars of white and red onions. Food Chem..

[35] Olsson M.E., Gustavsson K.-E., Vagen I.M. (2010). Quercetin and isorhamnetin in sweet and red cultivars of onion (*Allium cepa* L.) at harvest, after field curing, heat treatment, and storage. J. Agric. Food Chem..

[36] Tang X., Olatunji O.J., Zhou Y., Hou X. (2017). *Allium tuberosum*: Antidiabetic and hepatoprotective activities. Food Res. Int..

[37] Kim M.Y., Kim Y.C., Chung S.K. (2005). Identification and in vitro biological activities of flavonols in garlic leaf and shoot: Inhibition of soybean lipoxygenase and hyaluronidase activities and scavenging of free radicals. J. Sci. Food Agric..

[38] Nakane R., Iwashina T. (2015). Flavonol glycosides from the leaves of *Allium macrostemon*. Nat. Prod. Commun..

[39] Lin Y.-P., Lin L.-Y., Yeh H.-Y., Chuang C.-H., Tseng S.-W., Yen Y.-H. (2016). Antihyperlipidemic activity of *Allium chinense* bulbs. J. Food Drug Anal..

[40] Kothari D., Lee W.-D., Jung E.S., Niu K.-M., Lee C.H., Kim S.-K. (2020). Controlled Fermentation Using Autochthonous *Lactobacillus plantarum* improves antimicrobial potential of Chinese chives against poultry pathogens. Antibiotics.

[41] Arai Y., Watanabe S., Kimira M., Shimoi K., Mochizuki R., Kinae N. (2000). Dietary intakes of flavonols, flavones and isoflavones by Japanese women and the inverse correlation between quercetin intake and plasma LDL cholesterol concentration. J. Nutr..

[42] Olayeriju O.S., Olaleye M.T., Crown O.O., Komolafe K., Boligon A.A., Athayde M.L., Akindahunsi A.A. (2015). Ethylacetate extract of red onion (*Allium cepa* L.) tunic affects hemodynamic parameters in rats. Food Sci. Hum. Wellness.

[43] Carotenuto A., De Feo V., Fattorusso E., Lanzotti V., Magno S., Cicala C. (1996). The flavonoids of *Allium ursinum*. Phytochemistry.

[44] Corea G., Fattorusso E., Lanzotti V. (2003). Saponins and flavonoids of *Allium triquetrum*. J. Nat. Prod..

[45] Dziri S., Hassen I., Fatnassi S., Mrabet Y., Casabianca H., Hanchi B., Hosni K. (2012). Phenolic constituents, antioxidant and antimicrobial activities of rosy garlic (*Allium roseum* var. *odoratissimum*). J. Funct. Foods.

[46] Snoussi M., Trabelsi N., Dehmeni A., Benzekri R., Bouslama L., Hajlaoui B., Al-sieni A., Papetti A. (2016). Phytochemical analysis, antimicrobial and antioxidant activities of *Allium roseum* var. *odoratissimum* (Desf.) Coss extracts. Ind. Crop. Prod..

[47] Fattorusso E., Lanzotti V., Taglialatela-Scafati O., Cicala C. (2001). The flavonoids of leek, *Allium porrum*. Phytochemistry.

[48] Shi G.-Q., Yang J., Liu J., Liu S.-N., Song H.-X., Zhao W.-E., Liu Y.-Q. (2016). Isolation of flavonoids from onion skins and their effects on K562 cell viability. Bangladesh J. Pharmacol..

[49] Demirtas I., Erenler R., Elmastas M., Goktasoglu A. (2013). Studies on the antioxidant potential of flavones of *Allium vineale* isolated from its water-soluble fraction. Food Chem..

[50] Kim J., Kim J.-S., Park E. (2013). Cytotoxic and anti-inflammatory effects of onion peel extract on lipopolysaccharide stimulated human colon carcinoma cells. Food Chem. Toxicol..

[51] Bondonno N.P., Dalgaard F., Kyrø C., Murray K., Bondonno C.P., Lewis J.R., Croft K.D., Gislason G., Scalbert A., Cassidy A. (2019). Flavonoid intake is associated with lower mortality in the Danish Diet Cancer and Health Cohort. Nat. Commun..

[52] Rodríguez-García C., Sánchez-Quesada C., Gaforio J.J. (2019). Dietary flavonoids as cancer chemopreventive agents: An updated review of human studies. Antioxidants.

[53] Theodoratou E., Kyle J., Cetnarskyj R., Farrington S.M., Tenesa A., Barnetson R., Porteous M., Dunlop M., Campbell H. (2007). Dietary flavonoids and the risk of colorectal cancer. Cancer Epidemiol. Prev. Biomark..

[54] Lee W.S., Yi S.M., Yun J.W., Jung J.H., Kim D.H., Kim H.J., Chang S.-H., Kim G., Ryu C.H., Shin S.C. (2014). Polyphenols isolated from *Allium cepa* L. induces apoptosis by induction of p53 and suppression of Bcl-2 through inhibiting PI3K/Akt signaling pathway in AGS human cancer cells. J. Cancer Prev..

[55] Pan Y., Zheng Y.M., Ho W.S. (2018). Effect of quercetin glucosides from *Allium* extracts on HepG2, PC-3 and HT-29 cancer cell lines. Oncol. Lett..

[56] Myint A.A., Aregay M.G., Kang M., Kim B.-S., Lee Y.-W., Kim J. (2020). Comprehensive study on the formation mechanism of highly bioactive compounds from *Allium hookeri* root using subcritical water and their antioxidant and anticancer effects. J. Supercrit. Fluids.

[57] Grivennikov S.I., Greten F.R., Karin M. (2010). Immunity, inflammation, and cancer. Cell.

[58] Carmeliet P., Jain R.K. (2000). 2000. Angiogenesis in cancer and other diseases. Nature.

[59] Seyfi P., Mostafaie A., Mansouri K., Arshadi D., Mohammadi-Motlagh H.-R., Kiani A. (2010). In vitro and in vivo anti-angiogenesis effect of shallot (*Allium ascalonicum*): A heat-stable and flavonoid-rich fraction of shallot extract potently inhibits angiogenesis. Toxicol. In Vitro.

[60] Pi-Sunyer F.X. (2000). Obesity: Criteria and classification. Proc. Nutr. Soc..

[61] Kim O.Y., Lee S.M., Do H., Moon J., Lee K.H., Cha Y.J., Shin M.J. (2012). Influence of quercetin-rich onion peel extracts on adipokine expression in the visceral adipose tissue of rats. Phytother. Res..

[62] Moon J., Do H.-J., Kim O.Y., Shin M.-J. (2013). Antiobesity effects of quercetin-rich onion peel extract on the differentiation of 3T3-L1 preadipocytes and the adipogenesis in high fat-fed rats. Food Chem. Toxicol..

[63] Bae C.R., Park Y.K., Cha Y.S. (2014). Quercetin-rich onion peel extract suppresses adipogenesis by down-regulating adipogenic transcription factors and gene expression in 3T3-L1 adipocytes. J. Sci. Food Agric..

[64] Lee S.G., Parks J.S., Kang H.W. (2017). Quercetin, a functional compound of onion peel, remodels white adipocytes to brown-like adipocytes. J. Nutr. Biochem..

[65] Sung Y.-Y., Kim D.-S., Kim S.-H., Kim H.K. (2018). Aqueous and ethanolic extracts of welsh onion, *Allium fistulosum*, attenuate high-fat diet-induced obesity. BMC Complement. Altern. Med..

[66] Desjardins E.M., Steinberg G.R. (2018). Emerging role of AMPK in Brown and Beige adipose tissue (BAT): Implications for obesity, insulin resistance, and type 2 diabetes. Curr. Diabetes Rep..

[67] Nile A., Nile S.H., Kim D.H., Keum Y.S., Seok P.G., Sharma K. (2018). Valorization of onion solid waste and their flavonols for assessment of cytotoxicity, enzyme inhibitory and antioxidant activities. Food Chem. Toxicol..

[68] Zhou Y., Li C., Feng B., Chen B., Jin L., Shen Y. (2020). UPLC-ESI-MS/MS based identification and antioxidant, antibacterial, cytotoxic activities of aqueous extracts from storey onion (*Allium cepa* L. var. proliferum Regel). Food Res. Int..

[69] He Y., Jin H., Gong W., Zhang C., Zhou A. (2014). Effect of onion flavonoids on colorectal cancer with hyperlipidemia: An in vivo study. Oncotargets Ther..

[70] Kim K.-A., Yim J.-E. (2015). Antioxidative activity of onion peel extract in obese women: A randomized, double-blind, placebo-controlled study. J. Cancer Prev..

[71] Grzelak-Błaszczyk K., Milala J., Kosmala M., Kołodziejczyk K., Sójka M., Czarnecki A., Klewicki R., Juśkiewicz J., Fotschki B., Jurgoński A. (2018). Onion quercetin monoglycosides alter microbial activity and increase antioxidant capacity. J. Nutr. Biochem..

[72] Kim S.-H., Jo S.-H., Kwon Y.-I., Hwang J.-K. (2011). Effects of onion (*Allium cepa* L.) extract administration on intestinal α-glucosidases activities and spikes in postprandial blood glucose levels in SD rats model. Int. J. Mol. Sci..

[73] Jung J.Y., Lim Y., Moon M.S., Kim J.Y., Kwon O. (2011). Onion peel extracts ameliorate hyperglycemia and insulin resistance in high fat diet/streptozotocin-induced diabetic rats. Nutr. Metab..

[74] Ikechukwu O.J., Ifeanyi O.S. (2016). The antidiabetic effects of the bioactive flavonoid (kaempferol-3-O-β-d-6 {P-Coumaroyl} glucopyranoside) isolated from *Allium cepa*. Recent Pat. Anti-Infect. Drug Discov..

[75] Schulze C., Bangert A., Schwanck B., Vollert H., Blaschek W., Daniel H. (2015). Extracts and flavonoids from onion inhibit the intestinal sodium-coupled glucose transporter 1 (SGLT1) in vitro but show no anti-hyperglycaemic effects in vivo in normoglycaemic mice and human volunteers. J. Funct. Foods.

[76] Ni Z., Guo L., Liu F., Olatunji O.J., Yin M. (2019). *Allium tuberosum* alleviates diabetic nephropathy by supressing hyperglycemia-induced oxidative stress and inflammation in high fat diet/streptozotocin treated rats. Biomed. Pharmacother..

[77] Lee K.-H., Park E., Lee H.-J., Kim M.-O., Cha Y.-J., Kim J.-M., Lee H., Shin M.-J. (2011). Effects of daily quercetin-rich supplementation on cardiometabolic risks in male smokers. Nutr. Res. Pract..

[78] Hamauzu Y., Nosaka T., Ito F., Suzuki T., Torisu S., Hashida M., Fukuzawa A., Ohguchi M., Yamanaka S. (2011). Physicochemical characteristics of rapidly dried onion powder and its anti-atherogenic effect on rats fed high-fat diet. Food Chem..

[79] Lee S.-M., Moon J., Chung J.H., Cha Y.-J., Shin M.-J. (2013). Effect of quercetin-rich onion peel extracts on arterial thrombosis in rats. Food Chem. Toxicol..

[80] Nakayama H., Tsuge N., Sawada H., Higashi Y. (2013). Chronic intake of onion extract containing quercetin improved postprandial endothelial dysfunction in healthy men. J. Am. Coll. Nutr..

[81] Choi E.-Y., Lee H., Woo J.S., Jang H.H., Hwang S.J., Kim H.S., Kim W.-S., Kim Y.-S., Choue R., Cha Y.-J. (2015). Effect of onion peel extract on endothelial function and endothelial progenitor cells in overweight and obese individuals. Nutrition.

[82] Brüll V., Burak C., Stoffel-Wagner B., Wolffram S., Nickenig G., Müller C., Langguth P., Alteheld B., Fimmers R., Naaf S. (2015). Effects of a quercetin-rich onion skin extract on 24 h ambulatory blood pressure and endothelial function in overweight-to-obese patients with (pre-) hypertension: A randomised double-blinded placebo-controlled cross-over trial. Br. J. Nutr..

[83] Ro J.-Y., Ryu J.-H., Park H.-J., Cho H.-J. (2015). Onion (*Allium cepa* L.) peel extract has anti-platelet effects in rat platelets. Springerplus.

[84] Ko E.Y., Nile S.H., Jung Y.-S., Keum Y.S. (2018). Antioxidant and antiplatelet potential of different methanol fractions and flavonols extracted from onion (*Allium cepa* L.). 3 Biotech.

[85] Hwang I.K., Lee C.H., Yoo K.-Y., Choi J.H., Park O.K., Lim S.S., Kang I.-J., Kwon D.Y., Park J., Yi J.-S. (2009). Neuroprotective effects of onion extract and quercetin against ischemic neuronal damage in the gerbil hippocampus. J. Med. Food.

[86] Woo K.W., Moon E., Park S.Y., Kim S.Y., Lee K.R. (2012). Flavonoid glycosides from the leaves of *Allium victorialis* var. platyphyllum and their anti-neuroinflammatory effects. Bioorg. Med. Chem. Lett..

[87] Yang E.-J., Kim G.-S., Kim J.A., Song K.-S. (2013). Protective effects of onion-derived quercetin on glutamate-mediated hippocampal neuronal cell death. Pharmacogn. Mag..

[88] Lee B.K., Jung Y.-S. (2016). *Allium cepa* extract and quercetin protect neuronal cells from oxidative stress via PKC-ε inactivation/ERK1/2 activation. Oxidative Med. Cell. Longev..

[89] Singh T., Goel R.K. (2015). Neuroprotective effect of *Allium cepa* L. in aluminium chloride induced neurotoxicity. Neurotoxicology.

[90] Sarchielli E., Morelli A., Guarnieri G., Iorizzi M., Sgambati E. (2018). Neuroprotective effects of quercetin 4′-O-β-d-diglucoside on human striatal precursor cells in nutrient deprivation condition. Acta Histochem..

[91] Singh V., Krishan P., Shri R. (2018). Amelioration of ischaemia reperfusion-induced cerebral injury in mice by liposomes containing *Allium cepa* fraction administered intranasally. Artif. Cells Nanomed. Biotechnol..

[92] Kusminski C.M., Scherer P.E. (2012). Mitochondrial dysfunction in white adipose tissue. Trends Endocrinol. Metab..

[93] Henagan T., Cefalu W., Ribnicky D., Noland R., Dunville K., Campbell W., Stewart L., Forney L., Gettys T., Chang J. (2015). In vivo effects of dietary quercetin and quercetin-rich red onion extract on skeletal muscle mitochondria, metabolism, and insulin sensitivity. Genes Nutr..

[94] Grzelak-Błaszczyk K., Milala J., Kołodziejczyk K., Sójka M., Czarnecki A., Kosmala M., Klewicki R., Fotschki B., Jurgoński A., Juśkiewicz J. (2020). Protocatechuic acid and quercetin glucosides in onions attenuate changes induced by high fat diet in rats. Food Funct..

[95] Kim K.-A., Yim J.-E. (2016). The effect of onion peel extract on inflammatory mediators in korean overweight and obese women. Clin. Nutr. Res..

[96] World Health Organization (2016). Global Report on Diabetes: World Health Organization.

[97] Pantalone K.M., Hobbs T.M., Wells B.J., Kong S.X., Kattan M.W., Bouchard J., Yu C., Sakurada B., Milinovich A., Weng W. (2015). Clinical characteristics, complications, comorbidities and treatment patterns among patients with type 2 diabetes mellitus in a large integrated health system. BMJ Open Diabetes Res. Care.

[98] Nickerson H.D., Dutta S. (2012). Diabetic complications: Current challenges and opportunities. J. Cardiovasc. Transl. Res..

[99] Stolar M. (2010). Glycemic control and complications in type 2 diabetes mellitus. Am. J. Med..

[100] Shi G.-J., Li Y., Cao Q.-H., Wu H.-X., Tang X.-Y., Gao X.-H., Yu J.-Q., Chen Z., Yang Y. (2019). In vitro and in vivo evidence that quercetin protects against diabetes and its complications: A systematic review of the literature. Biomed. Pharmacother..

[101] Domingueti C.P., Dusse L.M.S.A., das Graças Carvalho M., de Sousa L.P., Gomes K.B., Fernandes A.P. (2016). Diabetes mellitus: The linkage between oxidative stress, inflammation, hypercoagulability and vascular complications. J. Diabetes Complicat..

[102] Elmarakby A.A., Sullivan J.C. (2012). Relationship between oxidative stress and inflammatory cytokines in diabetic nephropathy. Cardiovasc. Ther..

[103] Oguntibeju O.O. (2019). Type 2 diabetes mellitus, oxidative stress and inflammation: Examining the links. Int. J. Physiol. Pathophysiol. Pharmacol..

[104] Priya Rani M., Padmakumari K.P., Sankarikutty B., Lijo Cherian O., Nisha V.M., Raghu K.G. (2011). Inhibitory potential of ginger extracts against enzymes linked to type 2 diabetes, inflammation and induced oxidative stress. Int. J. Food Sci. Nutr..

[105] Song Y., Manson J.E., Buring J.E., Sesso H.D., Liu S. (2005). Associations of dietary flavonoids with risk of type 2 diabetes, and markers of insulin resistance and systemic inflammation in women: A prospective study and cross-sectional analysis. J. Am. Coll. Nutr..

[106] Moreira A.J., Fraga C., Alonso M., Collado P.S., Zetller C., Marroni C., Marroni N., González-Gallego J. (2004). Quercetin prevents oxidative stress and NF-κB activation in gastric mucosa of portal hypertensive rats. Biochem. Pharmacol..

[107] Alkhalidy H., Moore W., Zhang Y., McMillan R., Wang A., Ali M., Suh K.-S., Zhen W., Cheng Z., Jia Z. (2015). Small molecule kaempferol promotes insulin sensitivity and preserved pancreatic β-cell mass in middle-aged obese diabetic mice. J. Diabetes Res..

[108] Al-Numair K.S., Chandramohan G., Veeramani C., Alsaif M.A. (2015). Ameliorative effect of kaempferol, a flavonoid, on oxidative stress in streptozotocin-induced diabetic rats. Redox Rep..

[109] Zhang Y., Liu D. (2011). Flavonol kaempferol improves chronic hyperglycemia-impaired pancreatic beta-cell viability and insulin secretory function. Eur. J. Pharmacol..

[110] Lee Y.J., Suh K.S., Choi M.C., Chon S., Oh S., Woo J.T., Kim S.W., Kim J.W., Kim Y.S. (2010). Kaempferol protects HIT-T15 pancreatic beta cells from 2-deoxy-d-ribose-induced oxidative damage. Phytother. Res. Int. J. Devoted Pharmacol. Toxicol. Eval. Nat. Prod. Deriv..

[111] Chung M., Zhao N., Wang D., Shams-White M., Karlsen M., Cassidy A., Ferruzzi M., Jacques P.F., Johnson E.J., Wallace T.C. (2020). Dose-response relation between tea consumption and risk of cardiovascular disease and all-cause mortality: A systematic review and meta-analysis of population-based studies. Adv. Nutr..

[112] Raman G., Avendano E.E., Chen S., Wang J., Matson J., Gayer B., Novotny J.A., Cassidy A. (2019). Dietary intakes of flavan-3-ols and cardiometabolic health: Systematic review and meta-analysis of randomized trials and prospective cohort studies. Am. J. Clin. Nutr..

[113] Kim Y., Je Y. (2017). Flavonoid intake and mortality from cardiovascular disease and all causes: A meta-analysis of prospective cohort studies. Clin. Nutr. ESPEN.

[114] Hubbard G.P., Wolffram S., de Vos R., Bovy A., Gibbins J.M., Lovegrove J.A. (2006). Ingestion of onion soup high in quercetin inhibits platelet aggregation and essential components of the collagen-stimulated platelet activation pathway in man: A pilot study. Br. J. Nutr..

[115] Brüll V., Burak C., Stoffel-Wagner B., Wolffram S., Nickenig G., Müller C., Langguth P., Alteheld B., Fimmers R., Stehle P. (2017). Acute intake of quercetin from onion skin extract does not influence postprandial blood pressure and endothelial function in overweight-to-obese adults with hypertension: A randomized, double-blind, placebo-controlled, crossover trial. Eur. J. Nutr..

[116] Boland B., Yu W.H., Corti O., Mollereau B., Henriques A., Bezard E., Pastores G.M., Rubinsztein D.C., Nixon R.A., Duchen M.R. (2018). Promoting the clearance of neurotoxic proteins in neurodegenerative disorders of ageing. Nat. Rev. Drug Discov..

[117] Frandsen J., Narayanasamy P. (2017). Flavonoid enhances the glyoxalase pathway in cerebellar neurons to retain cellular functions. Sci. Rep..

[118] Lindqvist D., Dhabhar F.S., James S.J., Hough C.M., Jain F.A., Bersani F.S., Reus V.I., Verhoeven J.E., Epel E.S., Mahan L. (2017). Oxidative stress, inflammation and treatment response in major depression. Psychoneuroendocrinology.

[119] Fischer R., Maier O. (2015). Interrelation of oxidative stress and inflammation in neurodegenerative disease: Role of TNF. Oxidative Med. Cell. Longev..

[120] Reale M., Costantini E., Di Nicola M., D’Angelo C., Franchi S., D’Aurora M., Di Bari M., Orlando V., Galizia S., Ruggieri S. (2018). Butyrylcholinesterase and acetylcholinesterase polymorphisms in multiple sclerosis patients: Implication in peripheral inflammation. Sci. Rep..

[121] Nkpaa K.W., Onyeso G.I. (2018). Rutin attenuates neurobehavioral deficits, oxidative stress, neuro-inflammation and apoptosis in fluoride treated rats. Neurosci. Lett..

[122] Das U.N. (2007). Acetylcholinesterase and butyrylcholinesterase as possible markers of low-grade systemic inflammation. Med Sci. Monit..

[123] Khan M.T.H., Orhan I., Şenol F., Kartal M., Şener B., Dvorská M., Šmejkal K., Šlapetová T. (2009). Cholinesterase inhibitory activities of some flavonoid derivatives and chosen xanthone and their molecular docking studies. Chem.-Biol. Interact..

[124] Li Q., Wang Y., Mai Y., Li H., Wang Z., Xu J., He X. (2020). Health benefits of the flavonoids from onion: Constituents and their pronounced antioxidant and anti-neuroinflammatory capacities. J. Agric. Food Chem..

[125] Sharma D., Rani R., Chaturvedi M., Rohilla P., Yadav J. (2019). In silico and in vitro approach of *Allium cepa* and isolated quercetin against MDR bacterial strains and *Mycobacterium smegmatis*. S. Afr. J. Bot..

[126] Beceiro A., Tomás M., Bou G. (2013). Antimicrobial resistance and virulence: A successful or deleterious association in the bacterial world?. Clin. Microbiol. Rev..

[127] Gerdt J.P., Blackwell H.E. (2014). Competition studies confirm two major barriers that can preclude the spread of resistance to quorum-sensing inhibitors in bacteria. ACS Chem. Biol..

[128] Mahomoodally F., Ramcharun S., Zengin G. (2018). Onion and garlic extracts potentiate the efficacy of conventional antibiotics against standard and clinical bacterial isolates. Curr. Top. Med. Chem..

[129] Al-Yousef H.M., Ahmed A.F., Al-Shabib N.A., Laeeq S., Khan R.A., Rehman M.T., Alsalme A., Al-Ajmi M.F., Khan M.S., Husain F.M. (2017). Onion peel ethylacetate fraction and its derived constituent Quercetin 4′-O-β-D Glucopyranoside attenuates quorum sensing regulated virulence and biofilm formation. Front. Microbiol..

[130] Quecan B.X.V., Santos J.T., Rivera M.L., Hassimotto N.M., Almeida F.A., Pinto U.M. (2019). Effect of quercetin rich onion extracts on bacterial quorum sensing. Front. Microbiol..

[131] Farhadi L., Mohammadi-Motlagh H.-R., Seyfi P., Mostafaie A. (2014). Low concentrations of flavonoid-rich fraction of shallot extract induce delayed-type hypersensitivity and TH1 cytokine IFNγ expression in Balb/c Mice. Int. J. Mol. Cell. Med..

[132] Chen Y., Ding Z., Wu Y., Chen Q., Liu M., Yu H., Wang D., Zhang Y., Wang T. (2020). Effects of *Allium mongolicum* Regel and its flavonoids on constipation. Biomolecules.

[133] Cho Y.H., Lee J.W., Woo H.D., Lee S., Kim Y.J., Lee Y., Shin S., Joung H., Chung H.W. (2016). Protective effect of onion extract on bleomycin-induced cytotoxicity and genotoxicity in human lymphocytes. Int. J. Environ. Res. Public Health.

[134] Gansukh E., Nile A., Kim D.H., Oh J.W., Nile S.H. (2020). New insights into antiviral and cytotoxic potential of quercetin and its derivatives-A biochemical perspective. Food Chem..

[135] Pandey P., Rane J.S., Chatterjee A., Kumar A., Khan R., Prakash A., Ray S. (2020). Targeting SARS-CoV-2 spike protein of COVID-19 with naturally occurring phytochemicals: An in silico study for drug development. J. Biomol. Struct. Dyn..

[136] Schwarz S., Sauter D., Wang K., Zhang R., Sun B., Karioti A., Bilia A.R., Efferth T., Schwarz W. (2014). Kaempferol derivatives as antiviral drugs against the 3a channel protein of coronavirus. Planta Med..

[137] Sharifi-Rad M., Anil Kumar N.V., Zucca P., Varoni E.M., Dini L., Panzarini E., Rajkovic J., Tsouh Fokou P.V., Azzini E., Peluso I. (2020). Lifestyle, oxidative stress, and antioxidants: Back and forth in the pathophysiology of chronic diseases. Front. Physiol..

[138] Sies H., Jones D.P. (2020). Reactive oxygen species (ROS) as pleiotropic physiological signalling agents. Nat. Rev. Mol. Cell Biol..

[139] Liguori I., Russo G., Curcio F., Bulli G., Aran L., Della-Morte D., Gargiulo G., Testa G., Cacciatore F., Bonaduce D. (2018). Oxidative stress, aging, and diseases. Clin. Interv. Aging.

[140] Pizzino G., Irrera N., Cucinotta M., Pallio G., Mannino F., Arcoraci V., Squadrito F., Altavilla D., Bitto A. (2017). Oxidative Stress: Harms and benefits for human health. Oxidative Med. Cell. Longev..

[141] Hayes J.D., Dinkova-Kostova A.T. (2014). The Nrf2 regulatory network provides an interface between redox and intermediary metabolism. Trends Biochem. Sci..

[142] Cuadrado A., Rojo A.I., Wells G., Hayes J.D., Cousin S.P., Rumsey W.L., Attucks O.C., Franklin S., Levonen A.-L., Kensler T.W. (2019). Therapeutic targeting of the NRF2 and KEAP1 partnership in chronic diseases. Nat. Rev. Drug Discov..

[143] Nguyen T., Sherratt P.J., Nioi P., Yang C.S., Pickett C.B. (2005). Nrf2 controls constitutive and inducible expression of ARE-driven genes through a dynamic pathway involving nucleocytoplasmic shuttling by Keap1. J. Biol. Chem..

[144] Ahmed S.M.U., Luo L., Namani A., Wang X.J., Tang X. (2017). Nrf2 signaling pathway: Pivotal roles in inflammation. Biochim. Biophys. Acta (BBA)-Mol. Basis Dis..

[145] Pang S., Lynn D.A., Lo J.Y., Paek J., Curran S.P. (2014). SKN-1 and Nrf2 couples proline catabolism with lipid metabolism during nutrient deprivation. Nat. Commun..

[146] Chowdhry S., Zhang Y., McMahon M., Sutherland C., Cuadrado A., Hayes J.D. (2013). Nrf2 is controlled by two distinct β-TrCP recognition motifs in its Neh6 domain, one of which can be modulated by GSK-3 activity. Oncogene.

[147] Bryan H.K., Olayanju A., Goldring C.E., Park B.K. (2013). The Nrf2 cell defence pathway: Keap1-dependent and-independent mechanisms of regulation. Biochem. Pharmacol..

[148] Tanigawa S., Fujii M., Hou D.-X. (2007). Action of Nrf2 and Keap1 in ARE-mediated NQO1 expression by quercetin. Free Radic. Biol. Med..

[149] Yang J.H., Shin B.Y., Han J.Y., Kim M.G., Wi J.E., Kim Y.W., Cho I.J., Kim S.C., Shin S.M., Ki S.H. (2014). Isorhamnetin protects against oxidative stress by activating Nrf2 and inducing the expression of its target genes. Toxicol. Appl. Pharmacol..

[150] Pallauf K., Duckstein N., Hasler M., Klotz L.-O., Rimbach G. (2017). Flavonoids as putative inducers of the transcription factors Nrf2, FoxO, and PPARγ. Oxidative Med. Cell. Longev..

[151] Kashyap D., Sharma A., Tuli H.S., Sak K., Punia S., Mukherjee T.K. (2017). Kaempferol–A dietary anticancer molecule with multiple mechanisms of action: Recent trends and advancements. J. Funct. Foods.

[152] Hussein R.M., Mohamed W.R., Omar H.A. (2018). A neuroprotective role of kaempferol against chlorpyrifos-induced oxidative stress and memory deficits in rats via GSK3β-Nrf2 signaling pathway. Pestic. Biochem. Physiol..

[153] Sun L., Xu G., Dong Y., Li M., Yang L., Lu W. (2020). Quercetin protects against lipopolysaccharide-induced intestinal oxidative stress in broiler chickens through activation of Nrf2 pathway. Molecules.

[154] Zhang H., Sun S.-C. (2015). NF-κB in inflammation and renal diseases. Cell Biosci..

[155] Liu T., Zhang L., Joo D., Sun S.-C. (2017). NF-κB signaling in inflammation. Signal Transduct. Target. Ther..

[156] Morgan M.J., Liu Z.-g. (2011). Crosstalk of reactive oxygen species and NF-κB signaling. Cell Res..

[157] Espinosa L., Margalef P., Bigas A. (2015). Non-conventional functions for NF-κB members: The dark side of NF-κB. Oncogene.

[158] Ruiz P.A., Braune A., Hölzlwimmer G., Quintanilla-Fend L., Haller D. (2007). Quercetin inhibits TNF-induced NF-κ B transcription factor recruitment to proinflammatory gene promoters in murine intestinal epithelial cells. J. Nutr..

[159] Indra M.R., Karyono S., Ratnawati R., Malik S.G. (2013). Quercetin suppresses inflammation by reducing ERK1/2 phosphorylation and NF kappa B activation in leptin-induced human umbilical vein endothelial cells (HUVECs). BMC Res. Notes.

[160] Chekalina N., Burmak Y., Petrov Y., Borisova Z., Manusha Y., Kazakov Y., Kaidashev I. (2018). Quercetin reduces the transcriptional activity of NF-kB in stable coronary artery disease. Indian Heart J..

[161] Lee C.S. (2016). Flavonoid myricetin inhibits TNF-α-stimulated production of inflammatory mediators by suppressing the Akt, mTOR and NF-κB pathways in human keratinocytes. Eur. J. Pharmacol..

[162] Garg S., Malhotra R.K., Khan S.I., Sarkar S., Susrutha P., Singh V., Goyal S., Nag T.C., Ray R., Bhatia J. (2019). Fisetin attenuates isoproterenol-induced cardiac ischemic injury in vivo by suppressing RAGE/NF-κB mediated oxidative stress, apoptosis and inflammation. Phytomedicine.

[163] Qiu S., Sun G., Zhang Y., Li X., Wang R. (2016). Involvement of the NF-κB signaling pathway in the renoprotective effects of isorhamnetin in a type 2 diabetic rat model. Biomed. Rep..

[164] Lacroix M., Riscal R., Arena G., Linares L.K., Le Cam L. (2020). Metabolic functions of the tumor suppressor p53: Implications in normal physiology, metabolic disorders, and cancer. Mol. Metab..

[165] Beyfuss K., Hood D.A. (2018). A systematic review of p53 regulation of oxidative stress in skeletal muscle. Redox Rep..

[166] Clemente-Soto A.F., Salas-Vidal E., Milan-Pacheco C., Sánchez-Carranza J.N., Peralta-Zaragoza O., González-Maya L. (2019). Quercetin induces G2 phase arrest and apoptosis with the activation of p53 in an E6 expression-independent manner in HPV-positive human cervical cancer-derived cells. Mol. Med. Rep..

[167] Luo H., Rankin G.O., Li Z., DePriest L., Chen Y.C. (2011). Kaempferol induces apoptosis in ovarian cancer cells through activating p53 in the intrinsic pathway. Food Chem..

[168] Turrens J.F. (2003). Mitochondrial formation of reactive oxygen species. J. Physiol..

[169] Lagoa R., Graziani I., Lopez-Sanchez C., Garcia-Martinez V., Gutierrez-Merino C. (2011). Complex I and cytochrome care molecular targets of flavonoids that inhibit hydrogen peroxide production by mitochondria. Biochim. Biophys. Acta (BBA)-Bioenerg..

[170] Krauss S., Zhang C.-Y., Lowell B.B. (2005). The mitochondrial uncoupling-protein homologues. Nat. Rev. Mol. Cell Biol..

[171] Tarasov A.I., Griffiths E.J., Rutter G.A. (2012). Regulation of ATP production by mitochondrial Ca^2+^. Cell Calcium.

[172] Hamilton S., Terentyeva R., Kim T.Y., Bronk P., Clements R.T., O-Uchi J., Csordás G., Choi B.R., Terentyev D. (2018). Pharmacological modulation of mitochondrial Ca^2+^ content regulates sarcoplasmic reticulum Ca^2+^ release via oxidation of the ryanodine receptor by mitochondria-derived reactive oxygen species. Front. Physiol..

[173] Zheng H.-C. (2017). The molecular mechanisms of chemoresistance in cancers. Oncotarget.

[174] Steinberg G.R., Carling D. (2019). AMP-activated protein kinase: The current landscape for drug development. Nat. Rev. Drug Discov..

[175] Carbonell-Capella J.M., Buniowska M., Barba F.J., Esteve M.J., Frígola A. (2014). Analytical methods for determining bioavailability and bioaccessibility of bioactive compounds from fruits and vegetables: A review. Compr. Rev. Food Sci. Food Saf..

[176] Bohn T. (2014). Dietary factors affecting polyphenol bioavailability. Nutr. Rev..

[177] Williamson G., Kay C.D., Crozier A. (2018). The bioavailability, transport, and bioactivity of dietary flavonoids: A review from a historical perspective. Compr. Rev. Food Sci. Food Saf..

[178] Crozier A., Del Rio D., Clifford M.N. (2010). Bioavailability of dietary flavonoids and phenolic compounds. Mol. Asp. Med..

[179] Williamson G., Manach C. (2005). Bioavailability and bioefficacy of polyphenols in humans. II. Review of 93 intervention studies. Am. J. Clin. Nutr..

[180] Manach C., Williamson G., Morand C., Scalbert A., Rémésy C. (2005). Bioavailability and bioefficacy of polyphenols in humans. I. Review of 97 bioavailability studies. Am. J. Clin. Nutr..

[181] DuPont M.S., Day A.J., Bennett R.N., Mellon F.A., Kroon P.A. (2004). Absorption of kaempferol from endive, a source of kaempferol-3-glucuronide, in humans. Eur. J. Clin. Nutr..

[182] Guo Y., Bruno R.S. (2015). Endogenous and exogenous mediators of quercetin bioavailability. J. Nutr. Biochem..

[183] Crespy V., Morand C., Besson C., Manach C., Demigne C., Remesy C. (2002). Quercetin, but not its glycosides, is absorbed from the rat stomach. J. Agric. Food Chem..

[184] Hollman P.C., de Vries J.H., van Leeuwen S.D., Mengelers M.J., Katan M.B. (1995). Absorption of dietary quercetin glycosides and quercetin in healthy ileostomy volunteers. Am. J. Clin. Nutr..

[185] Wiczkowski W., Romaszko J., Bucinski A., Szawara-Nowak D., Honke J., Zielinski H., Piskula M.K. (2008). Quercetin from shallots (*Allium cepa* L. var. aggregatum) is more bioavailable than its glucosides. J. Nutr..

[186] Day A.J., Mellon F., Barron D., Sarrazin G., Morgan M.R., Williamson G. (2001). Human metabolism of dietary flavonoids: Identification of plasma metabolites of quercetin. Free Radic. Res..

[187] Nemeth K., Piskula M. (2007). Food content, processing, absorption and metabolism of onion flavonoids. Crit. Rev. Food Sci. Nutr..

[188] Hollman P.C., Bijsman M.N., Van Gameren Y., Cnossen E.P., De Vries J.H., Katan M.B. (1999). The sugar moiety is a major determinant of the absorption of dietary flavonoid glycosides in man. Free Radic. Res..

[189] Olthof M.R., Hollman P.C., Vree T.B., Katan M.B. (2000). Bioavailabilities of quercetin-3-glucoside and quercetin-4′-glucoside do not differ in humans. J. Nutr..

[190] Lee J., Mitchell A.E. (2012). Pharmacokinetics of quercetin absorption from apples and onions in healthy humans. J. Agric. Food Chem..

[191] Lesser S., Cermak R., Wolffram S. (2004). Bioavailability of quercetin in pigs is influenced by the dietary fat content. J. Nutr..

[192] Guo Y., Mah E., Davis C.G., Jalili T., Ferruzzi M.G., Chun O.K., Bruno R.S. (2013). Dietary fat increases quercetin bioavailability in overweight adults. Mol. Nutr. Food Res..

[193] Mullen W., Edwards C.A., Crozier A. (2006). Absorption, excretion and metabolite profiling of methyl-, glucuronyl-, glucosyl-and sulpho-conjugates of quercetin in human plasma and urine after ingestion of onions. Br. J. Nutr..

[194] Gowd V., Karim N., Shishir M.R.I., Xie L., Chen W. (2019). Dietary polyphenols to combat the metabolic diseases via altering gut microbiota. Trends Food Sci. Technol..

[195] Wang W., Sun C., Mao L., Ma P., Liu F., Yang J., Gao Y. (2016). The biological activities, chemical stability, metabolism and delivery systems of quercetin: A review. Trends Food Sci. Technol..

[196] Cardona F., Andrés-Lacueva C., Tulipani S., Tinahones F.J., Queipo-Ortuño M.I. (2013). Benefits of polyphenols on gut microbiota and implications in human health. J. Nutr. Biochem..

[197] Manach C., Scalbert A., Morand C., Rémésy C., Jiménez L. (2004). Polyphenols: Food sources and bioavailability. Am. J. Clin. Nutr..

[198] Zhao J., Yang J., Xie Y. (2019). Improvement strategies for the oral bioavailability of poorly water-soluble flavonoids: An overview. Int. J. Pharm..

[199] Chen Z.-p., Sun J., Chen H.-x., Xiao Y.-y., Liu D., Chen J., Cai H., Cai B.-c. (2010). Comparative pharmacokinetics and bioavailability studies of quercetin, kaempferol and isorhamnetin after oral administration of *Ginkgo biloba* extracts, *Ginkgo biloba* extract phospholipid complexes and *Ginkgo biloba* extract solid dispersions in rats. Fitoterapia.

[200] Khalilzadeh M.A., Borzoo M. (2016). Green synthesis of silver nanoparticles using onion extract and their application for the preparation of a modified electrode for determination of ascorbic acid. J. Food Drug Anal..

[201] Peñalva R., Esparza I., Morales-Gracia J., Gonzalez-Navarro C.J., Larrañeta E., Irache J.M. (2019). Casein nanoparticles in combination with 2-hydroxypropyl-β-cyclodextrin improves the oral bioavailability of quercetin. Int. J. Pharm..

[202] Ahmad N., Ahmad R., Naqvi A.A., Alam M.A., Ashafaq M., Abdur Rub R., Ahmad F.J. (2018). Intranasal delivery of quercetin-loaded mucoadhesive nanoemulsion for treatment of cerebral ischaemia. Artif. Cells Nanomed. Biotechnol..

[203] Zhao G., Duan J., Xie Y., Lin G., Luo H., Li G., Yuan X. (2013). Effects of solid dispersion and self-emulsifying formulations on the solubility, dissolution, permeability and pharmacokinetics of isorhamnetin, quercetin and kaempferol in total flavones of *Hippophae rhamnoides* L.. Drug Dev. Ind. Pharm..

[204] Sun M., Gao Y., Pei Y., Guo C., Li H., Cao F., Yu A., Zhai G. (2010). Development of nanosuspension formulation for oral delivery of quercetin. J. Biomed. Nanotechnol..

[205] Forte L., Torricelli P., Boanini E., Gazzano M., Rubini K., Fini M., Bigi A. (2016). Antioxidant and bone repair properties of quercetin-functionalized hydroxyapatite: An in vitro osteoblast–osteoclast–endothelial cell co-culture study. Acta Biomater..

[206] Mulholland P., Ferry D., Anderson D., Hussain S., Young A., Cook J., Hodgkin E., Seymour L., Kerr D. (2001). Pre-clinical and clinical study of QC12, a water-soluble, pro-drug of quercetin. Ann. Oncol..

[207] Vissiennon C., Nieber K., Kelber O., Butterweck V. (2012). Route of administration determines the anxiolytic activity of the flavonols kaempferol, quercetin and myricetin—Are they prodrugs?. J. Nutr. Biochem..

[208] Bahram-Parvar M., Lim L.T. (2018). Fresh-cut onion: A review on processing, health benefits, and shelf-life. Compr. Rev. Food Sci. Food Saf..

[209] Rohn S., Buchner N., Driemel G., Rauser M., Kroh L.W. (2007). Thermal degradation of onion quercetin glucosides under roasting conditions. J. Agric. Food Chem..

[210] Rodrigues A., Pérez-Gregorio M., García-Falcón M., Simal-Gándara J. (2009). Effect of curing and cooking on flavonols and anthocyanins in traditional varieties of onion bulbs. Food Res. Int..

[211] Makris D.P., Rossiter J.T. (2001). Domestic processing of onion bulbs (*Allium cepa*) and asparagus spears (*Asparagus officinalis*): Effect on flavonol content and antioxidant status. J. Agric. Food Chem..

[212] Lee S.U., Lee J.H., Choi S.H., Lee J.S., Ohnisi-Kameyama M., Kozukue N., Levin C.E., Friedman M. (2008). Flavonoid content in fresh, home-processed, and light-exposed onions and in dehydrated commercial onion products. J. Agric. Food Chem..

[213] Bhatta S., Stevanovic Janezic T., Ratti C. (2020). Freeze-drying of plant-based foods. Foods.

[214] Pérez-Gregorio M., Regueiro J., González-Barreiro C., Rial-Otero R., Simal-Gándara J. (2011). Changes in antioxidant flavonoids during freeze-drying of red onions and subsequent storage. Food Control.

[215] Bisakowski B., Atwal A.S., Gardner N., Champagne C.P. (2007). Effect of lactic acid fermentation of onions (*Allium cepa*) on the composition of flavonol glucosides. Int. J. Food Sci. Technol..

[216] Yang E.J., Kim S.I., Park S.Y., Bang H.Y., Jeong J.H., So J.H., Rhee I.K., Song K.S. (2012). Fermentation enhances the in vitro antioxidative effect of onion (*Allium cepa*) via an increase in quercetin content. Food Chem. Toxicol..

[217] Chung D.-M., Chung Y.-C., Maeng P.J., Chun H.-K. (2011). Regioselective deglycosylation of onion quercetin glucosides by *Saccharomyces cerevisiae*. Biotechnol. Lett..

[218] Lee Y.G., Cho J.Y., Kim Y.M., Moon J.H. (2016). Change in flavonoid composition and antioxidative activity during fermentation of onion (*Allium cepa* L.) by *Leuconostoc mesenteroides* with different salt concentrations. J. Food Sci..

[219] Millet A.S., Lamy E., Jonas D., Stintzing F., Mersch-Sundermann V., Merfort I. (2012). Fermentation enhances the biological activity of *Allium cepa* bulb extracts. J. Agric. Food Chem..

[220] Świeca M., Gawlik-Dziki U., Dziki D., Baraniak B., Czyż J. (2013). The influence of protein–flavonoid interactions on protein digestibility in vitro and the antioxidant quality of breads enriched with onion skin. Food Chem..

[221] Piechowiak T., Grzelak-Błaszczyk K., Bonikowski R., Balawejder M. (2020). Optimization of extraction process of antioxidant compounds from yellow onion skin and their use in functional bread production. LWT.

[222] Świeca M., Gawlik-Dziki U. (2015). Nutritional and health-promoting properties of bean paste fortified with onion skin in the light of phenolic–food matrix interactions. Food Funct..

[223] Sung Y.-Y., Kim S.-H., Kim D.-S., Park S.H., Yoo B.W., Kim H.K. (2014). Nutritional composition and anti-obesity effects of cereal bar containing *Allium fistulosum* (welsh onion) extract. J. Funct. Foods.

[224] Zhao Y., Fan D., Zheng Z.P., Li E.T., Chen F., Cheng K.W., Wang M. (2017). 8-C-(E-phenylethenyl) quercetin from onion/beef soup induces autophagic cell death in colon cancer cells through ERK activation. Mol. Nutr. Food Res..

[225] Zmora N., Suez J., Elinav E. (2019). You are what you eat: Diet, health and the gut microbiota. Nat. Rev. Gastroenterol. Hepatol..

